# Syk-dependent tyrosine phosphorylation of 3BP2 is required for optimal FcRγ-mediated phagocytosis and chemokine expression in U937 cells

**DOI:** 10.1038/s41598-017-11915-5

**Published:** 2017-09-13

**Authors:** Kazuyasu Chihara, Yuji Kato, Hatsumi Yoshiki, Kenji Takeuchi, Shigeharu Fujieda, Kiyonao Sada

**Affiliations:** 1 Department of Genome Science and Microbiology, Faculty of Medical Sciences, University of Fukui, 23-3 Matsuoka-Shimoaizuki, Eiheiji, Fukui 910-1193 Japan; 2 Life Science Innovation Center, University of Fukui, 3-9-1 Bunkyo, Fukui, Fukui, 910-8507 Japan; 3 Department of Otorhinolaryngology Head & Neck Surgery, Faculty of Medical Sciences, University of Fukui, 23-3 Matsuoka-Shimoaizuki, Eiheiji, Fukui 910-1193 Japan

## Abstract

The adaptor protein c-Abl SH3 domain binding protein-2 (3BP2) is tyrosine phosphorylated by Syk in response to cross-linking of antigen receptors, which in turn activates various immune responses. Recently, a study using the mouse model of cherubism, a dominant inherited disorder caused by mutations in the gene encoding 3BP2, showed that 3BP2 is involved in the regulation of phagocytosis mediated by Fc receptor for IgG (FcγR) in macrophages. However, the molecular mechanisms underlying 3BP2-mediated regulation of phagocytosis and the physiological relevance of 3BP2 tyrosine phosphorylation remains elusive. In this study, we established various gene knockout U937 cell lines using the CRISPR/Cas9 system and found that 3BP2 is rapidly tyrosine phosphorylated by Syk in response to cross-linking of FcγRI. Depletion of 3BP2 caused significant reduction in the Fc receptor γ chain (FcRγ)-mediated phagocytosis in addition to the FcγRI-mediated induction of chemokine mRNA for IL-8, CCL3L3 and CCL4L2. Syk-dependent tyrosine phosphorylation of 3BP2 was required for overcoming these defects. Finally, we found that the PH and SH2 domains play important roles on FcγRI-mediated tyrosine phosphorylation of 3BP2 in HL-60 cells. Taken together, these results indicate that Syk-dependent tyrosine phosphorylation of 3BP2 is required for optimal FcRγ-mediated phagocytosis and chemokine expression.

## Introduction

Myeloid phagocytic cells such as monocytes, macrophages, dendritic cells and neutrophils are known to play important roles in the clearance of invading pathogens by the process called phagocytosis^1, 2^. It is widely accepted that recognition of pathogenic particles by phagocytic receptors expressed on the cell surface is the first step to trigger a variety of cellular responses, including internalisation of particles into phagosomes and production of inflammatory cytokines and chemokines^2^. Among a number of phagocytic receptors, the molecular features of Fc receptors for IgG (FcγRs) have been extensively studied^3–5^. In humans, FcγRI and FcγRIIIA form a protein complex with an immunoreceptor tyrosine-based activation motif (ITAM) bearing adaptor, known as Fc receptor γ chain (FcRγ). In addition, FcγRIIA and FcγRIIC are known to possess intramolecular ITAM in the cytoplasmic region. Cross-linking of these receptors induces tyrosine phosphorylation of ITAM through Src-type kinases such as Hck, Lyn and Fgr, leading to the recruitment of Syk for activation^6, 7^. Activation of Syk is critical for engulfment of pathogens and production of cytokines and chemokines in response to cross-linking of FcγRs^8^. In addition to Src-type kinases, it has been shown that Abl family kinases contribute to FcγR- and complement receptor-mediated phagocytosis through regulation of Syk activity^9^.

Numerous studies have established that Syk is critical for immune responses mediated by various antigen receptors such as the B-cell receptor (BCR) and high-affinity IgE receptor (FcεRI), in addition to FcγRs^8, 10, 11^. Moreover, recent studies have uncovered that Syk also regulates CARD9-Malt1-BCL10 signalling^12^ and NLRP3 inflammasome activation^13^ in innate immune responses.

In this study, we investigated the role of an adaptor protein, c-Abl Src homology (SH) 3 domain binding protein-2 (3BP2), on Syk-mediated cellular signalling. The 3BP2 protein was originally identified as an Abl-binding protein of unknown function^14^. Human 3BP2 is a 561 amino acid protein which consists of an N-terminal pleckstrin homology (PH) domain, a proline-rich region which interacts with the SH3 domain of Abl and a C-terminal SH2 domain^15–17^. 3BP2 is rapidly tyrosine phosphorylated in response to antigen receptor cross-linking on mast cells^18, 19^, B cells^20–22^, T cells^23^ and natural killer cells^24^. An *in vitro* experiment using COS7 cells demonstrated that Syk, Lyn and Btk phosphorylated 3BP2 but Pyk2 and FAK could not^19^. Of these, we found that Syk predominantly phosphorylates Tyr174, 183 and 448 (446 in mouse protein) of 3BP2^19^. Previously, we have shown that phosphorylation of Tyr183 of 3BP2 is important for association with phospholipase C (PLC) γ2 and Vav1, leading to BCR- and T cell receptor-mediated activation of nuclear factor of activated T cells (NFAT)^21, 23^. Studies using 3BP2-knockout (KO) mice revealed that 3BP2 is required for optimal BCR-mediated activation of B cells^25, 26^.

In addition to its role with immune receptor signalling, genetic studies have shown that 3BP2 is responsible for the dominant inherited disorder cherubism, which is characterised by excessive bone resorption in the jaw bones^16^. Using a mouse model of cherubism, in which the most frequent mutation in patients (a substitution of Pro418 to Arg) was introduced into the mouse gene, it has been shown that the homozygous mutation causes severe bone loss. This is because of an increased number of macrophages with enhanced production of tumour necrosis factor (TNF)-α and large osteoclasts with high bone-resorbing activity^27, 28^. Biochemical analyses have revealed that the cherubism mutation causes increased expression of the 3BP2 protein because of the loss of recognition by Tankyrase, a poly (ADP-ribose) polymerase which facilitates the proteasome-mediated degradation of 3BP2^29, 30^. Accumulation of 3BP2 protein is believed to induce the activation of Src, Vav and Syk, accompanied with enhanced production of TNF-α in macrophages and an increase in osteoclast formation^27, 29^.

Recently, it was reported that phagocytic activity and the production of inflammatory cytokines were both reduced in macrophages derived from 3BP2-KO mice and enhanced in those derived from cherubism mutant mice^31^. In addition, another study has shown that stimulation of TLR2 or TLR4 induces Syk-dependent tyrosine phosphorylation of 3BP2 in macrophages^32^. Although these lines of evidences imply that Syk-dependent tyrosine phosphorylation of 3BP2 plays an important role in FcγR-mediated signalling in phagocytic cells, the molecular details remain obscure.

In this study, we established various KO cell lines derived from human monocytic U937 cells by using the CRISPR/Cas9 system^33^. Using these cells, we investigated the role of 3BP2 on FcRγ-mediated phagocytosis and gene expression and analysed the physiological relevance of the tyrosine phosphorylation of 3BP2.

## Results

### Cross-linking of FcγRI induces Syk-dependent tyrosine phosphorylation of 3BP2 in U937 cells

To investigate the role of 3BP2 in FcγR-mediated signalling, we established various KO cell lines by using the CRISPR/Cas9 system in U937 cells. To express the Cas9 protein along with a single-guide RNA, which is responsible for recognition of the target site, the oligo DNAs (Supplementary Table S1) were synthesised and ligated into the pX330 vector^33^. The resultant plasmid was transfected into parental U937 cells and transfected cells were screened by G418.

**Figure 1:**
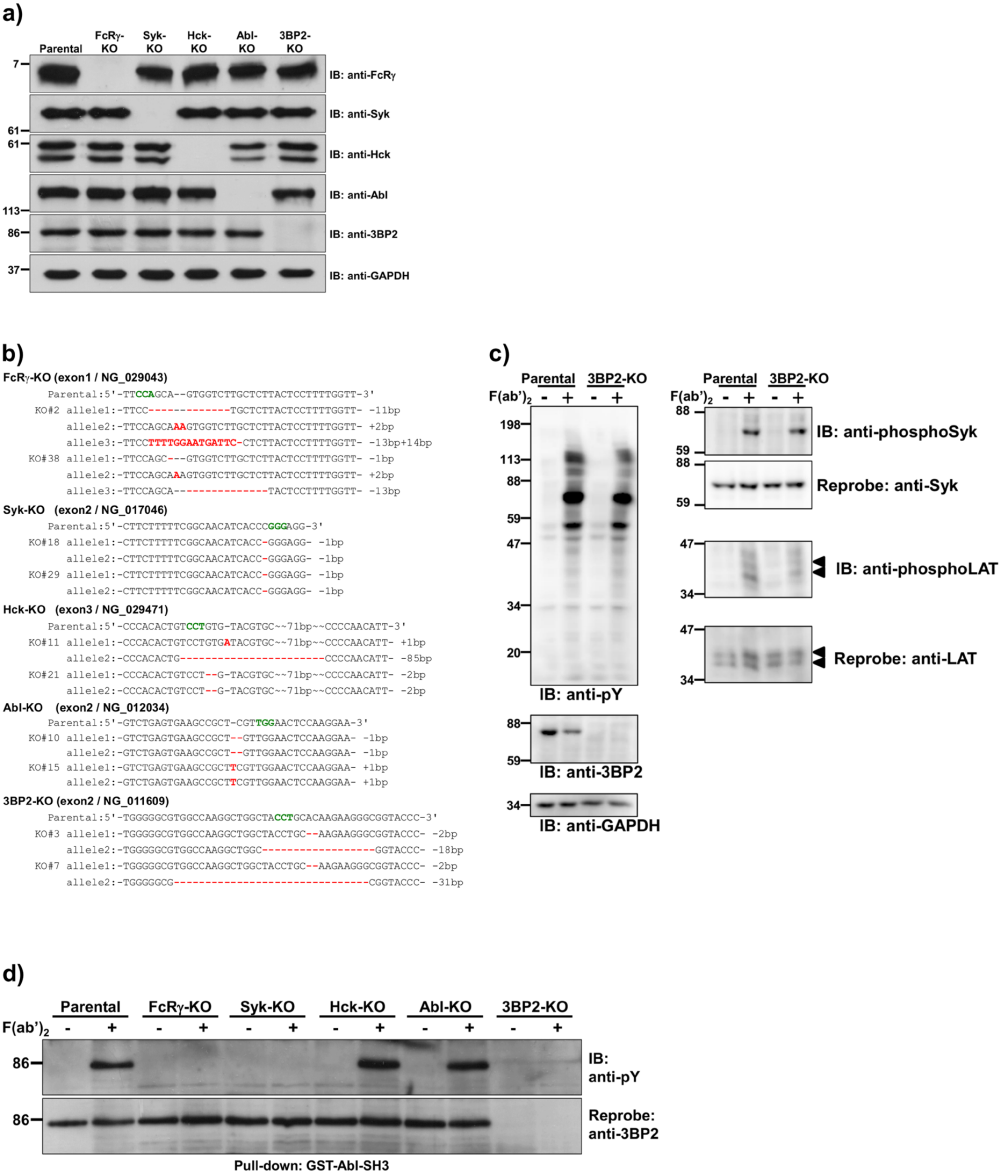
Syk-dependent tyrosine phosphorylation of 3BP2 in the FcγRI-stimulated cells. (**a**) Knockout (KO) cells used in this study. Protein lysates, obtained from U937 (Parental) or KO cells in which the indicated gene was targeted, were separated by SDS-PAGE and analysed by immunoblotting. (**b**) The mutations generated by the CRISPR/Cas9 system in the target genes. The mutations were confirmed via sequencing. Targeted exon and GenBank accession number of the indicated gene are shown in parenthesis. Characters in red or green indicate the replaced nucleotides or the protospacer adjacent motif, respectively. Dash indicates a gap between nucleotides. (**c**) FcγRI-mediated tyrosine phosphorylation of cellular proteins. After the indicated cells were stimulated without (−) or with (+) cross-linking of FcγRI (F(ab′)_2_), cell lysates were prepared and proteins were separated by SDS-PAGE and analysed by immunoblotting. Arrowheads indicate the position of LAT. (**d**) Tyrosine phosphorylation of 3BP2 induced by cross-linking of FcγRI. After the cells were stimulated without (−) or with (+) cross-linking of FcγRI (F(ab′)_2_), cell lysates were prepared and subjected to a pull-down assay. Proteins bound to GST tagged Abl SH3 domain (GST-Abl-SH3) were separated by SDS-PAGE and analysed by immunoblotting. (**a**,**c** and **d**) Molecular size markers are indicated on the left in kDa. Data are representative of three independent experiments. Full-length blots are presented in Supplementary Fig. S1. Two cloned cell lines for each gene were established and similar results were obtained from both cell lines (Supplementary Fig. S2).

As shown in Figs 1a and S1, expression of target genes in the selected clones was analysed by immunoblotting. Although we established at least two of each clone in which expression of the FcRγ, Syk, Hck, Abl, or 3BP2 protein was abrogated, the expression level of Abl protein was relatively low in Hck-KO#21 (Supplementary Fig. S2). Genomic DNA was extracted from each clone and sequencing analyses were performed to identify mutations generated by the CRISPR/Cas9 system. As shown in Fig. 1b, insertion or deletion mutations, of which most cause a frameshift in translation, were found at all alleles of each gene in the established clones. In the case of gene encoding FcRγ, we found that three alleles exist in U937 cells. Of note, we established a clone (3BP2-KO#3) which has a capacity to express a six amino acid truncated form of 3BP2 because of an 18 base pair deletion in one allele, although expression of the protein was abrogated (lane 6, Fig. 1a).

It is well established that expression of FcγRI on U937 cells is dramatically increased by stimulation with interferon-γ (IFN-γ)^34, 35^. After sensitised with monomeric mouse IgG2a (mIgG2a) whose Fc portion is specifically recognised by FcγRI^36^, the cells were stimulated with F(ab′)_2_ fragments of anti-mouse IgG to trigger the cross-linking of FcγRI. As shown in Fig. 1c, the cross-linking of FcγRI dramatically increased the tyrosine phosphorylation level of cellular proteins including Syk (Tyr525/526) and linker for activation of T cells (LAT) (Tyr191) in both parental U937 and 3BP2-KO cells, although it was reported that the silencing of 3BP2 reduces tyrosine phosphorylation level of Syk and LAT in FcεRI-stimulated mast cells^37^. These results suggest that 3BP2 is not required for the phosphorylation of Syk and LAT induced by cross-linking of FcγRI. We next examined whether cross-linking of FcγRI induced tyrosine phosphorylation of 3BP2 in IFN-γ stimulated U937 cells. Because we could not efficiently immunoprecipitate human 3BP2 using antibodies which are commercially available, we performed a pull-down assay using a GST-tagged Abl-SH3 domain (GST-Abl-SH3) which interacts with the proline-rich region of 3BP2^14^. As shown in Fig. 1d, a pull-down assay using GST-Abl-SH3 followed by immunoblotting analyses demonstrated that cross-linking of FcγRI significantly increased the tyrosine phosphorylation of 3BP2 in parental U937 cells. Phosphorylation of 3BP2 in the FcRγ- or Syk-KO cells was undetectable, as observed in the 3BP2-KO cells. We also found that KO of Hck or Abl had almost no effect on the level of tyrosine phosphorylation. Taken together, these results indicated that cross-linking of FcγRI induces Syk-dependent tyrosine phosphorylation of 3BP2 in IFN-γ stimulated U937 cells.

### Role of tyrosine kinases on FcRγ-mediated phagocytosis

**Figure 2:**
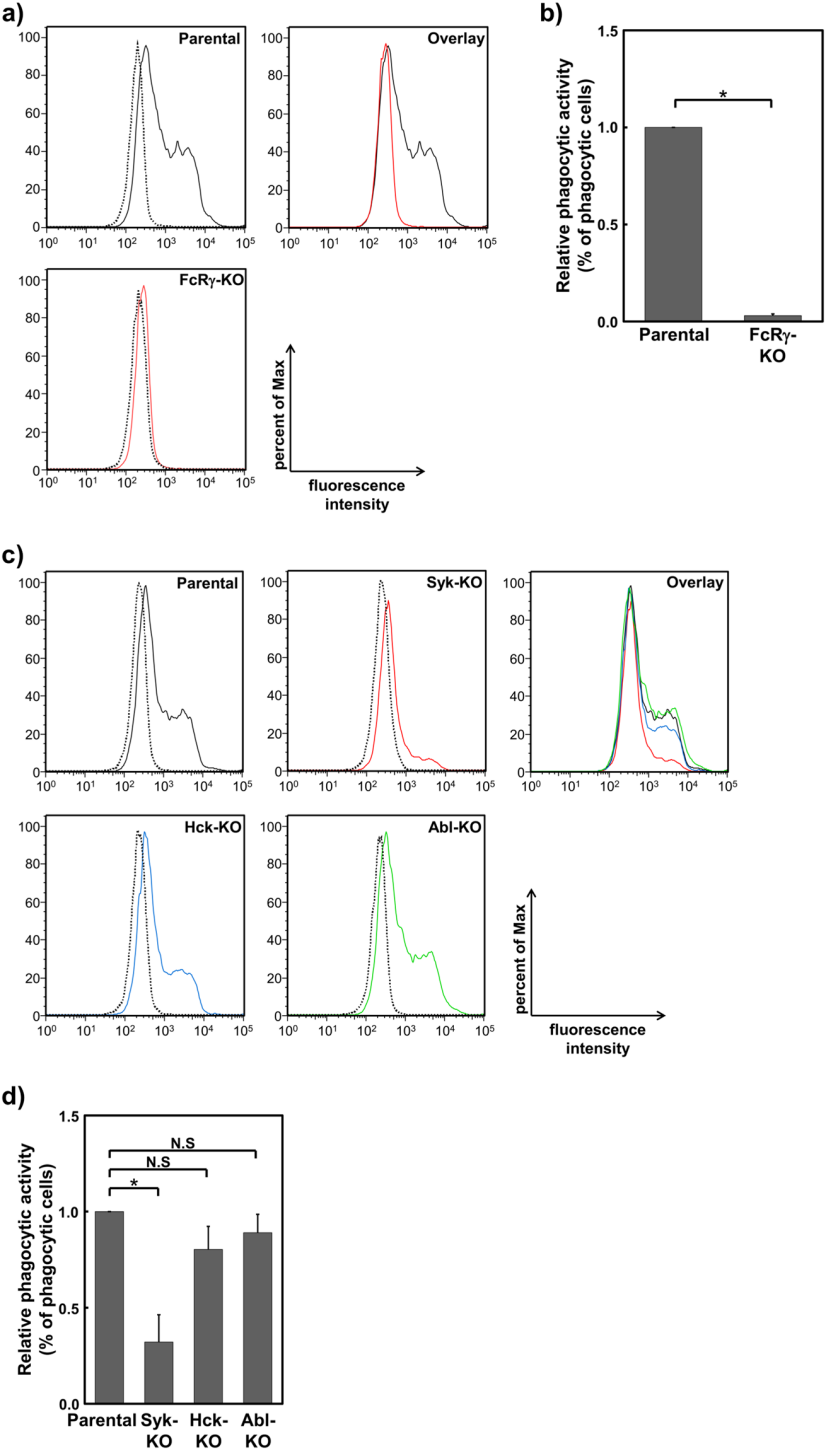
Syk is critical for FcRγ-mediated phagocytosis. (**a** and **c**) Phagocytic activity in U937 (Parental) and knockout (KO) cells in which the indicated gene was targeted. After the cells were incubated with opsonised zymosan for 0 min (dotted line) or 60 min (solid line), the fluorescence intensity of the cells was measured by flow cytometry. Histograms from each cell line were overlaid (Overlay) to compare the phagocytic activities. Data are representative of three independent experiments. Similar results were obtained from both replicate cell lines (Supplementary Fig. S2). (**b** and **d**) Quantitative analyses of relative phagocytic activity. The percentage of zymosan positive cells (% of phagocytic cells) was compared with data obtained from the Parental cells. The relative values obtained from the indicated cells are expressed as relative phagocytic activity. Results are shown as the mean ± SD of three independent experiments. Similar results were obtained from both replicate cell lines. *P < 0.01 was considered statistically significant. N.S. = not significant.

It is well established that FcRγ is essential for phagocytosis of antibody-coated particles in macrophages^38^. To investigate the role of 3BP2 on FcRγ-mediated phagocytosis, we first confirmed the requirement of FcRγ for engulfment of opsonised zymosan in IFN-γ-stimulated U937 cells. As shown in Fig. 2a, flow cytometric analyses showed that fluorescein labelled opsonised zymosan was efficiently engulfed by parental U937 cells, whereas this was not observed in FcRγ-KO cells. The data were further quantitatively analysed to calculate and compare the percentage of zymosan positive cells with those observed in parental U937 cells. As shown in Fig. 2b, a dramatic reduction in the proportion of zymosan positive cells was observed in the FcRγ-KO cells.

Next, we analysed the phagocytic activity of Syk-, Hck- and Abl-KO cells. As observed in FcRγ-KO cells, phagocytic activity and the percentage of zymosan positive cells was dramatically reduced in Syk-KO cells (Fig. 2c and d). Although phagocytic activity in the Hck- and Abl-KO cells was slightly reduced, it was not statistically significant. Taken together, these results suggested that Syk is critical for the FcRγ-mediated phagocytosis of opsonised zymosan on U937 cells and absence of Hck or Abl causes no significant defect of phagocytic activity.

### Effect of Syk-dependent tyrosine phosphorylation of 3BP2 and the cherubism mutation on FcRγ-mediated phagocytosis

**Figure 3:**
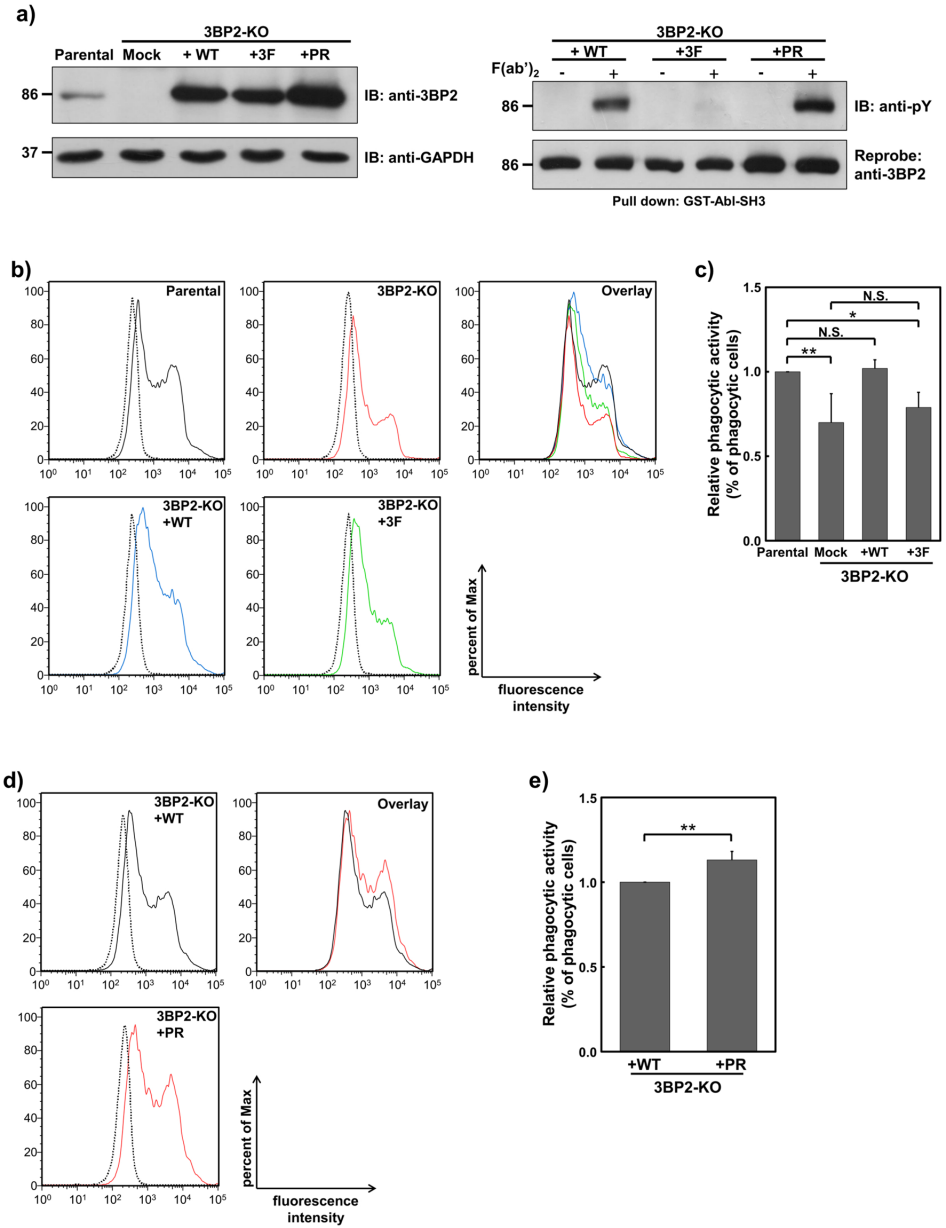
Effect of Syk-dependent tyrosine phosphorylation of 3BP2 and the cherubism mutation on FcRγ-mediated phagocytosis. (**a**) Establishment of 3BP2-knockout (KO) cells stably expressing HA-tagged 3BP2 (left panel). Protein lysates obtained from U937 (Parental) cells, 3BP2-KO cells, 3BP2-KO cells expressing HA-3BP2-WT (3BP2-KO+WT), HA-3BP2-3F (3BP2-KO+3F) or HA-3BP2-PR (3BP2-KO+PR) were separated by SDS-PAGE and analysed by immunoblotting. Tyrosine phosphorylation of HA-3BP2 induced by cross-linking of FcγRI (right panel). After the cells were stimulated without (−) or with (+) cross-linking of FcγRI (F(ab′)_2_), cell lysates were prepared and subjected to a pull-down assay. Proteins bound to GST tagged Abl SH3 domain (GST-Abl-SH3) were separated by SDS-PAGE and analysed by immunoblotting. Molecular size markers are indicated on the left in kDa. Data are representative of three independent experiments. Full-length blots are presented in Supplementary Fig. S1. Two cloned cells expressing each form of HA-3BP2 were established and similar results were obtained from both cell lines (Supplementary Fig. S2). (**b** and **d**) Phagocytic activity in the indicated cells. After the cells were incubated with opsonised zymosan for 0 min (dotted line) or 60 min (solid line), the fluorescence intensity of the cells was measured by flow cytometry. Histograms from each cell line were overlaid (Overlay) to compare phagocytic activity. Data are representative of three independent experiments. Similar results were obtained from both replicate cell lines (Supplementary Fig. S2). (**c** and **e**) Quantitative analyses of relative phagocytic activity in the indicated cells. The percentage of zymosan positive cells (% of phagocytic cells) was compared with data obtained from the Parental cells (**c**), or that obtained from the 3BP2-KO + WT cells (**e**). The relative values obtained from indicated cells are expressed as relative phagocytic activity. Results are shown as the mean ± SD of three independent experiments. Similar results were obtained from both replicate cell lines. *P < 0.01 and **P < 0.05 were considered statistically significant. N.S. = not significant.

Phosphorylation of Tyr174, 183 and 448 in 3BP2 plays an important role in antigen receptor mediated signal transduction^19–24^. In addition, substitution of Pro418 to Arg in human 3BP2 results in a gain-of-function mutation that leads to a genetic disorder called cherubism^16, 27^. To investigate the requirement for 3BP2 in FcRγ-mediated phagocytosis and the physiological relevance of tyrosine phosphorylation of 3BP2 and the cherubism mutation, we established 3BP2-KO cells stably expressing the following: HA-tagged 3BP2 wild-type (HA-3BP2-WT); HA-3BP2 whose Tyr174, 183, and 448 were substituted to Phe (HA-3BP2-3F); HA-3BP2 whose Pro418 was substituted to Arg (HA-3BP2-PR). The expression level of all HA-3BP2 proteins in the 3BP2-KO cells was higher than that of endogenous 3BP2 in the parental U937 cells, with expression of HA-3BP2-PR the highest (Fig. 3a, left panel). We next evaluated the FcγRI-mediated tyrosine phosphorylation of HA-3BP2 using a pull-down assay. As shown in the right panel of Fig. 3a, the levels of tyrosine phosphorylation of HA-3BP2-WT and HA-3BP2-PR, but not HA-3BP2-3F, were dramatically increased in response to the cross-linking of FcγRI. As Syk was essential for FcγRI-mediated tyrosine phosphorylation of 3BP2 in U937 cells (Fig. 1c), it would be reasonable to consider that Syk is critical for the phosphorylation of Tyr174, 183 and 448 in U937 cells. Of note, the level of tyrosine phosphorylated HA-3BP2-PR was relatively higher than that of HA-3BP2-WT.

Using these established cells, we next analysed the role of 3BP2 on FcRγ-mediated phagocytosis. As shown in Fig. 3b, phagocytic activity in the 3BP2-KO cells was significantly decreased when compared with the parental U937 cells. In contrast, phagocytic activity in the 3BP2-KO cells expressing HA-3BP2-WT was comparable with the parental U937 cells, suggesting that expression of 3BP2 is important for optimal FcRγ-mediated phagocytosis. Interestingly, we found that phagocytic activity in the 3BP2-KO cells expressing HA-3BP2-3F is not significantly different to that observed in the 3BP2-KO cells. Quantitative analyses indicated that expression of HA-3BP2-WT, but not HA-3BP2-3F, is effective for the recovery of decreased phagocytic activity observed in the 3BP2-KO cells (Fig. 3c). Taken together, these results suggested that 3BP2 plays an important role in FcRγ-mediated phagocytosis and Syk-dependent tyrosine phosphorylation is critical for the regulation by 3BP2.

Recently, it has been shown that the cherubism mutation causes increased phagocytic activity towards both opsonised and non-opsonised particles^31^. Thus, we further analysed the effects of the cherubism mutation on the regulation of phagocytic activity by 3BP2. Phagocytic activity in the 3BP2-KO cells expressing HA-3BP2-PR was significantly higher than that observed in the HA-3BP2-WT expressing cells (Fig. 3d and e). Consistent with the findings of Prod’Homme *et al*.^31^, our results suggested that the cherubism mutation results in an up-regulation of the 3BP2-mediated phagocytosis of opsonised zymosan.

### 3BP2 plays an important role in Syk-dependent gene expression induced by cross-linking of FcγRI

**Figure 4:**
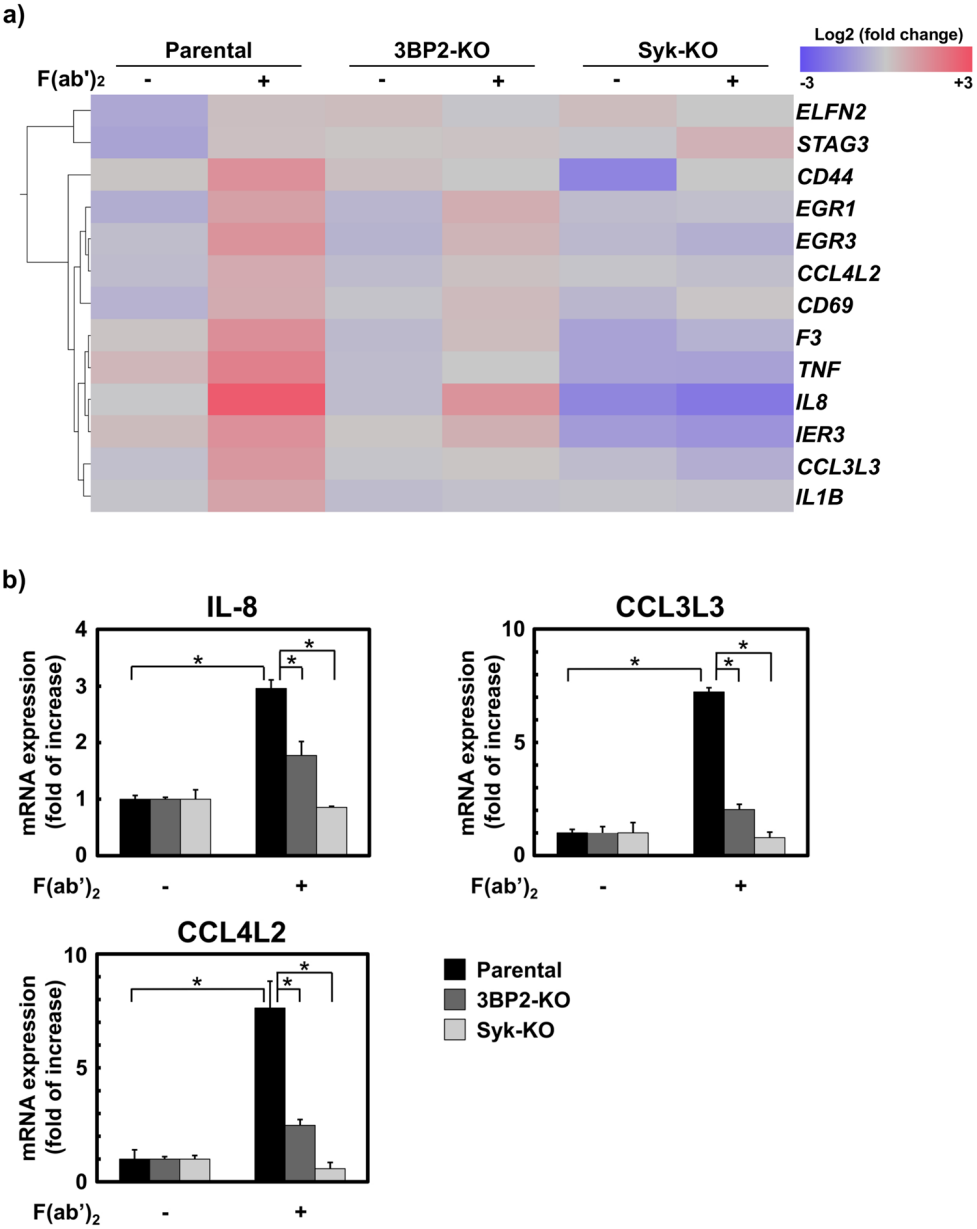
3BP2 is critical for chemokine mRNA expression induced by cross-linking of FcγRI. U937 (Parental), Syk-knockout (Syk-KO) and 3BP2-knockout (3BP2-KO) cells were stimulated without (−) or with (+) the cross-linking of FcγRI (F(ab′)_2_) for 60 min. Total RNA was recovered from these cell lines. (**a**) Heat map of genes whose expression was increased more than 2-fold in the Parental cells in response to the cross-linking of FcγRI. Expression levels are coloured blue for low intensity and red for high intensity. The gene symbols are indicated on the right. Fold changes observed in each cell line are listed online in Supplementary Table S2. (**b**) Quantitative analyses of chemokine mRNA expression induced by the stimulation of FcγRI. Real-time PCR was performed to quantitate mRNA expression for IL-8, CCL3L3 and CCL4L2 in the Parental, Syk-KO and 3BP2-KO cells stimulated without (−) or with (+) the cross-linking of FcγRI (F(ab′)_2_). The expression relative to that observed in unstimulated cells was shown. Data are representative of three independent experiments and are presented as the mean ± SD. Similar results were obtained using both replicate cell lines (Supplementary Fig. S2). *P < 0.01 was considered statistically significant.

It has been shown that FcγRI-mediated signalling plays an important role in regulating gene expression of various cytokines and chemokines^39, 40^. Therefore, we performed microarray analysis to identify genes whose expression is regulated by 3BP2. As shown in Fig. 4a, we observed up-regulation of various genes in parental U937 cells in response to cross-linking of FcγRI. The up-regulation of most of these genes, except for *ELFN2*, *STAG3* and *CD44*, was partially reduced in 3BP2-KO cells and dramatically suppressed in Syk-KO cells, suggesting that 3BP2 is involved in FcγRI-mediated gene expression under the control of Syk.

Interestingly, we found that the expression of mRNA for chemokine IL-8, in addition to CCL3L3 and CCL4L2, was reduced in FcγRI-stimulated 3BP2-KO cells when compared with the parental U937 cells. CCL3L3 and CCL4L2 are known as a variant of human macrophage inflammatory protein (MIP)-α and MIP-β, respectively^41^. To evaluate the microarray data, we further analysed the expression of mRNA for these chemokines by quantitative real-time PCR. As shown in Fig. 4b, the expression of IL-8, CCL3L3 and CCL4L2 mRNA was significantly up-regulated in parental U937 cells in response to cross-linking of FcγRI, whereas no up-regulation was evident in the Syk-KO cells. In this experimental system, up-regulation of these genes was significantly reduced in the 3BP2-KO cells. Taken together, these results demonstrated that 3BP2 plays an important role in the regulation of Syk-dependent chemokine expression induced by cross-linking of FcγRI.

### Effect of Syk-dependent tyrosine phosphorylation of 3BP2 and the cherubism mutation on FcγRI-mediated chemokine expression

**Figure 5:**
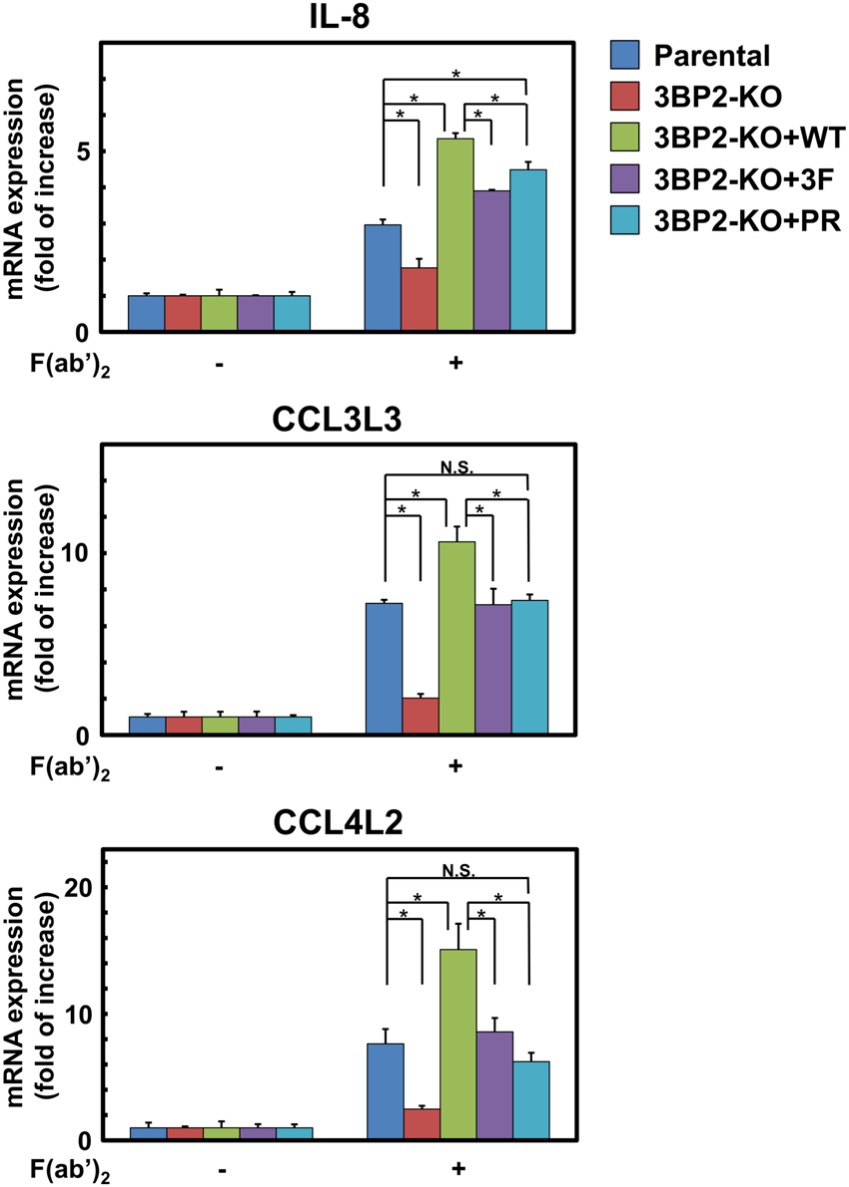
Effect of Syk-dependent tyrosine phosphorylation of 3BP2 and the cherubism mutation on chemokine mRNA expression induced by cross-linking of FcγRI. U937 (Parental) cells, 3BP2-knockout (3BP2-KO) cells, 3BP2-knockout cells expressing HA-3BP2-WT (3BP2-KO+WT), HA-3BP2-3F (3BP2-KO + 3F) and HA-3BP2-PR (3BP2-KO+PR) were stimulated without (−) or with (+) the cross-linking of FcγRI (F(ab′)_2_) for 60 min. After total RNA was recovered and reverse transcribed, real-time PCR was performed to quantitate mRNA expression for IL-8, CCL3L3 and CCL4L2. The expression relative to that observed in unstimulated cells was shown. Data are representative of three independent experiments and are presented as the mean ± SD. Similar results were obtained from both replicate cell lines (Supplementary Fig. S2). *P < 0.01 was considered statistically significant. N.S. = not significant.

Given that the FcγRI-mediated up-regulation of chemokine mRNA was significantly reduced in 3BP2-KO cells, we next analysed the physiological relevance of tyrosine phosphorylation of 3BP2 and the cherubism mutation. As shown in Fig. 5, we found that the induction of IL-8, CCL3L3, and CCL4L2 mRNAs in 3BP2-KO cells expressing HA-3BP2-WT was higher than that observed in the 3BP-KO and parental U937 cells. This suggests that overexpression of HA-3BP2-WT up-regulates Syk-dependent chemokine mRNA expression in 3BP2-KO cells. In contrast, the up-regulation observed in the 3BP2-KO cells expressing HA-3BP2-3F was lower than that observed in the HA-3BP2-WT expressing cells. Taken together, these results suggested that Syk-dependent tyrosine phosphorylation of 3BP2 plays an important role in the regulation of FcγRI-mediated chemokine mRNA expression. Regarding the effects of the cherubism mutation, we found that induction of IL-8 mRNA observed in the 3BP2-KO cells expressing HA-3BP2-PR is higher than that observed in the parental U937 cells. However, this effect was not significant in the case of CCL3L3 or CCL4L2. Therefore, it was likely that the cherubism mutation influences the signal transduction downstream of 3BP2 to regulate Syk-dependent chemokine mRNA expression.

### The PH and SH2 domains play important roles on the tyrosine phosphorylation of 3BP2 induced by cross-linking of FcγRI

**Figure 6:**
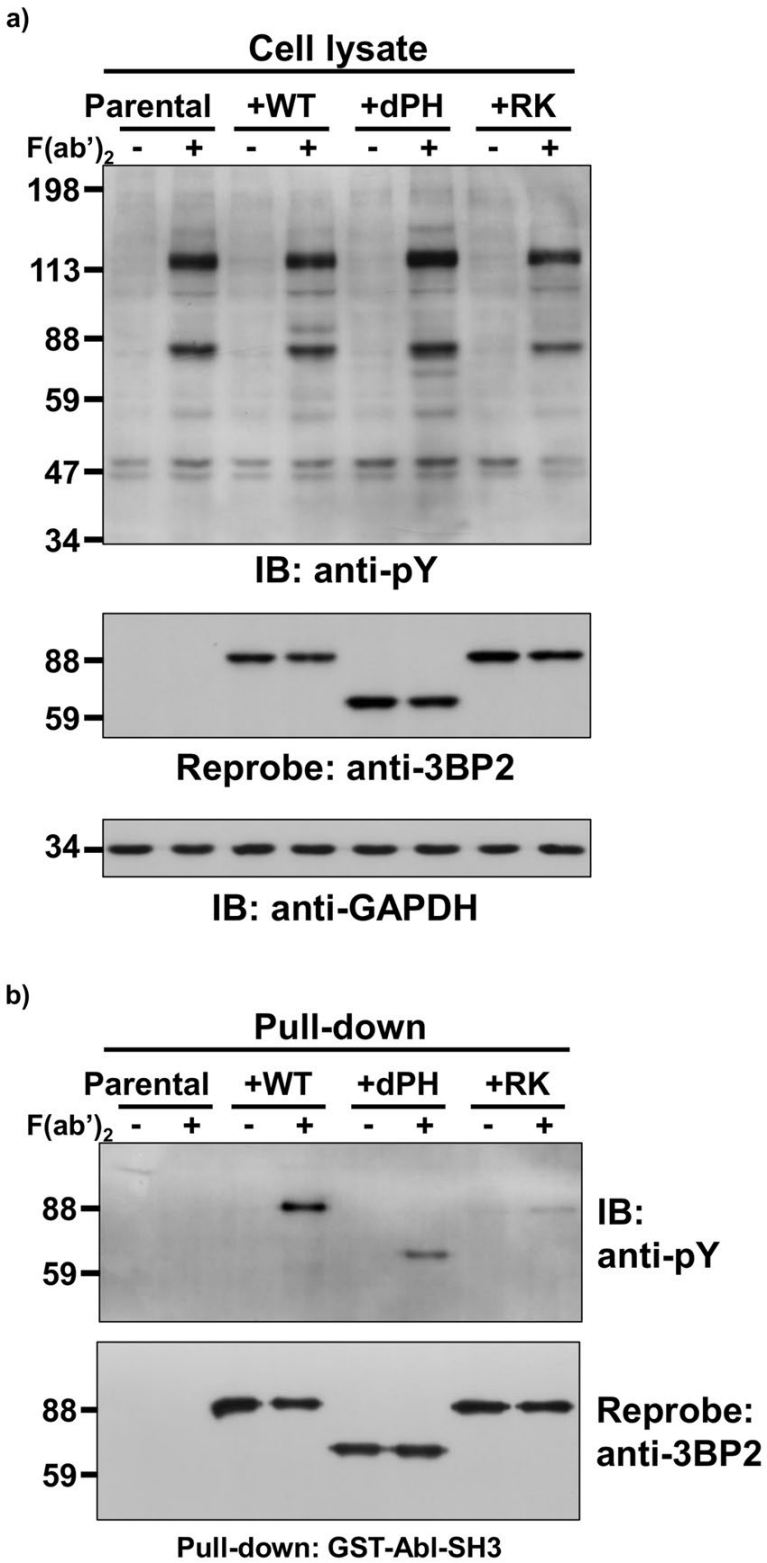
Roles of the PH and SH2 domains on the tyrosine phosphorylation of 3BP2 induced by cross-linking of FcγRI. FcγRI-induced tyrosine phosphorylation of cellular proteins in HL-60 cells stably expressing various mutant forms of 3BP2. Parental HL-60 cells (Parental), HL-60 cells stably expressing HA-3BP2-WT (+WT), HA-3BP2-dPH (+dPH) and HA-3BP2-RK (+RK) were stimulated without (−) or with (+) cross-linking of FcγRI (F(ab′)_2_). Cell lysates were prepared and subjected to a pull-down assay. Cell lysates (**a**) or proteins bound to GST tagged Abl SH3 domain (GST-Abl-SH3) (**b**) were separated by SDS-PAGE and analysed by immunoblotting. (**a** and **b**) Molecular size markers are indicated on the left in kDa. Data are representative of three independent experiments.

3BP2 was identified as Syk kinases-interacting protein-2 (SKIP-2) by yeast two-hybrid system and the SH2 domain of 3BP2 was shown to interact with Syk^15^. In addition, it has been shown that the PH and SH2 domains are required for the 3BP2-mediated activation of NFAT in BCR-activated human B cell lines^20^. We have also shown that the SH2 domain of 3BP2 plays important roles on the tyrosine phosphorylation of 3BP2 in BCR-activated human and chicken B cell lines^21, 22^. Because the data obtained in this study indicated that the tyrosine phosphorylation of 3BP2 play important roles on FcγRI-mediated phagocytosis and chemokine expression, we examined whether the PH and SH2 domains of 3BP2 are also involved in the tyrosine phosphorylation of cellular proteins in FcγRI-signalling. HL-60 cells are known to express FcγRI on the cell surface^42^ and the expression level of 3BP2 was relatively low when compared with U937 cells (data not shown). As other human model, we established HL-60 cells stably overexpressing HA-3BP2-WT, HA-3BP2 whose PH domain is deleted (HA-3BP2-dPH) or HA-3BP2 whose Arg488 is substituted to Lys for abrogating the function of SH2 domain (HA-3BP2-RK)^18, 21, 22^. Consistent with the data shown in Fig. 1c, the tyrosine phosphorylation of cellular proteins induced by cross-linking of FcγRI was comparable between parental HL-60 cells and cells overexpressing HA-3BP2-WT (Fig. 6a). Although the tyrosine phosphorylation of HA-3BP2-WT was dramatically induced by cross-linking of FcγRI, the phosphorylation level was partially reduced in HA-3BP2-dPH, and dramatically abrogated in HA-3BP2-RK when compared with that observed in HA-3BP2-WT (Fig. 6b). Taken together, these results suggest that the SH2 domain in addition to PH domain of 3BP2 play important roles on FcγRI-mediated tyrosine phosphorylation of 3BP2.

## Discussion

In this study, we established various gene KO cell lines by using the CRISPR/Cas9 system. Regarding the establishment of the 3BP2-KO cells, we found that the six amino acid deletion in the PH domain abrogated expression of the 3BP2 protein in one of the established cell lines (3BP2-KO#3) (Fig. 1a and b). It is important to note that the two amino acid deletion in the PH domain also caused a similar result in the other cloned cells (data not shown). Previously, it was reported that a 37 amino acid deletion in the highly conserved region of Cbl proteins almost completely abrogates expression of the c-Cbl protein in mice^43^. The PH domain of 3BP2 is also highly conserved beyond vertebrate species. Thus, it is possible that the deletion of particular amino acids may reduce the stability of the 3BP2 protein.

We found that cross-linking of FcγRI induces Syk-dependent tyrosine phosphorylation of endogenous 3BP2 (Fig. 1d). It has been shown that the Src-type kinases Hck, Lyn and Fgr are critical for tyrosine phosphorylation of FcRγ in mouse macrophages^7^. In addition, Hck and Lyn form a protein complex with FcγRI and are activated in response to receptor cross-linking on human monocytic cells^6^. On the other hand, the Abl family kinases Abl and Arg are activated by cross-linking of FcγRs to modulate the kinase activity of Syk^9^. Although these observations raise a possibility that 3BP2 is directly tyrosine-phosphorylated by Hck or Abl, the absence of these kinases had no effect on the tyrosine phosphorylation level of 3BP2. As we have shown that Lyn has a potential to phosphorylate 3BP2^19^, there is a possibility that Lyn phosphorylates 3BP2 in FcγRI-stimulated cells. However, we could not analyse the role of Lyn in this study because we failed to establish Lyn-KO cells. None the less, considering the result showing that the tyrosine phosphorylation of 3BP2 is completely abrogated in the absence of Syk (Fig. 1d) and our previous studies^19, 21, 22^, we propose that 3BP2 is tyrosine phosphorylated by Syk in FcγRI-stimulated cells.

As observed in mouse macrophages^38^, the phagocytic activity of FcRγ-KO cells was completely abrogated (Fig. 2a and b). In addition to FcγRs, it is known that integrin CD11b/CD18 (also known as Mac-I) induces complement-mediated phagocytosis^44^. Thus, we established CD18-KO cells to examine the role of Mac-I on the phagocytosis of opsonised zymosan. However, we found that an absence of CD18 had no effect on phagocytosis (data not shown). Therefore, it is likely that phagocytosis of opsonised zymosan is dominantly mediated by FcγRs, specifically FcγRI, which is known to be highly expressed on IFN-γ-stimulated U937 cells^34, 35^.

It was reported that interaction with 3BP2 enhances the activity of the Src-type kinases in addition to Abl and Syk. For instance, we reported that Syk-dependent phosphorylation of Tyr448 (446 in mouse 3BP2) is recognised by the SH2 domain of Lyn^19^. Another study found that the proline-rich region of 3BP2 acts as a ligand for the SH3 domain of Abl in osteoblasts^45^. It was also demonstrated that the SH2 domain of 3BP2 interacts with Syk^15^. In fact, it has been shown that the deletion or loss of function mutation of SH2 domain causes the dramatic reduction of tyrosine phosphorylation (Fig. 6b) and functional defect of 3BP2^15, 20–22^. As these kinases are known to play an important role in FcRγ-mediated phagocytosis^7, 9^, there is a possibility that an absence of 3BP2 influences their kinase activity, which in turn decreases the phagocytic activity. However, we found that an absence of Hck or Abl had no significant effect on phagocytic activity (Fig. 2c). Furthermore, we did not observe any significant change in the levels of FcγRI-mediated tyrosine phosphorylation of cellular proteins including Syk (Tyr525/526 are located within the activation loop of the kinase domain), between parental U937 cells and 3BP2-KO cells (Fig. 1c). Similar results were obtained using FcγRI-stimulated HL-60 cells and cells overexpressing 3BP2 (Fig. 6a). Therefore, it is likely that the reduced phagocytic activity observed in the 3BP2-KO cells (Fig. 3b and c) is not because of altered Hck, Abl or Syk activity caused by an absence of 3BP2. Instead, 3BP2 might regulate phagocytosis through modulating the downstream signalling of Syk.

Although expression of 3BP2 in the 3BP2-KO cells expressing HA-3BP2 was higher than that observed in parental U937 cells, only expression of HA-3BP2-3F was unable to restore the defect in phagocytic activity in the 3BP2-KO cells (Fig. 3b and c). The results indicate that Syk-dependent phosphorylation of Tyr174, 183 or 448 plays an important role in the regulation of phagocytic activity by 3BP2. It was demonstrated that 3BP2 interacts with the PLCγ and Vav family proteins^20, 24^ and phosphorylation of Tyr183 is important for the interaction^21, 24^. In fact, we found that PLCγ2 and Vav1 bound to the synthetic phospho-peptide whose sequence is identical with that around Tyr183 of 3BP2^21^ (Supplementary Fig. S3). The PLCγ and Vav family proteins are believed to be downstream molecules of Syk and involved in FcRγ-mediated phagocytosis by regulating calcium mobilisation and the actin cytoskeleton^46^. Therefore, Syk-dependent tyrosine phosphorylation of 3BP2 might facilitate the interaction with the PLCγ and Vav family proteins, which in turn promotes FcRγ-mediated phagocytosis.

In addition to these signalling molecules, we have shown that the SH2 domain of 3BP2 interacts with adaptor proteins such as LAT and B cell linker protein (BLNK) to activate antigen receptor-mediated signalling in mast cells and B cells^18, 21^. Importantly, it was reported that LAT is tyrosine phosphorylated in response to cross-linking of FcγRI (Fig. 1c) and is involved in FcγR-mediated phagocytosis^47^. In addition, BLNK is also tyrosine phosphorylated in response to FcγRI stimulation^48^. Therefore, it is possible that interaction with these adaptor molecules is necessary for the 3BP2-dependent regulation of signalling pathways, even if overexpression of the non-tyrosine phosphorylated form of 3BP2 is insufficient for the restoration of phagocytic activity in 3BP2-KO cells.

The cherubism mutation is believed to increase the stability of the 3BP2 protein, which leads to accumulation of 3BP2 in macrophages or osteoclasts and activation of signalling molecules such as the Src-type kinases, Vav and Syk^27, 29, 31^. In this study, we found that the expression level of HA-3BP2-PR was relatively higher than that of HA-3BP2-WT and the amount of tyrosine phosphorylated 3BP2 in 3BP2-KO cells correlated well with their expression levels (Fig. 3a). Furthermore, we found that the phagocytic activity observed in 3BP2-KO cells expressing HA-3BP2-PR was higher than that observed in 3BP2-KO cells expressing HA-3BP2-WT (Fig. 3d and e). This implies that the increased stability of 3BP2 generated by the cherubism mutation results in accumulation of the tyrosine phosphorylated form of 3BP2 in FcRγ-stimulated cells, thus promoting phagocytic activity.

In addition to phagocytosis, microarray analysis revealed that 3BP2 regulates Syk-dependent gene expression in FcγRI-stimulated cells (Fig. 4a). It was reported that the transcription factors early growth response 1 and 3 are up-regulated in cells stimulated by the C-type lectin family proteins Dectin-1^49^ and Mincle^50^. Another C-type lectin protein, CD69, is a cell surface protein expressed on activated leukocytes^51^. Regarding the expression of TNF-α and IL-1β, it has been shown that 3BP2 is involved in their regulation in macrophages^27, 32^. In addition to these genes, we found that 3BP2 participates in the Syk-dependent up-regulation of chemokines IL-8, CCL3L3, and CCL4L2 mRNAs (Fig. 4a and b), suggesting that 3BP2 might play an important role in regulating chemokine expression to recruit leukocytes to the site of pathogen invasion. Given that the mRNA induction of these chemokines in 3BP2-KO cells expressing HA-3BP2-WT was higher than that of HA-3BP2-3F expressing cells, Syk-dependent tyrosine phosphorylation of 3BP2 positively regulates the chemokine expression induced by the cross-linking of FcγRI (Fig. 5). Interestingly, in contrast to phagocytosis, overexpression of HA-3BP2-3F partially overcame the defect in chemokine expression in the 3BP2-KO cells. This suggests that in addition to Syk-dependent tyrosine phosphorylation, the SH2 domain or proline-rich region of 3BP2 might also have the potential to enhance chemokine expression through interaction with other signalling molecules.

Although we found that the cherubism mutation results in increased IL-8 mRNA expression when compared with levels in the parental U937 cells, the effect was not observed with CCL3L3 and CCL4L2 mRNA expression. More importantly, the induction of chemokine mRNAs observed in 3BP2-KO cells expressing HA-3BP2-PR was lower than those observed in HA-3BP2-WT expressing cells. Previously, we have shown that overexpression of cherubism mutant forms of 3BP2 in RBL-2H3 cells resulted in reduced cytokine mRNA expression induced by FcεRI stimulation^52^. In addition, zymosan-induced expression of TNF-α in macrophages derived from heterozygous cherubism mutant mice was found to be higher than those derived from homozygous mutant mice^31^. Therefore, we hypothesise that the effects of the cherubism mutation may vary between signalling pathways and the type of cell, rather than solely leading to activation of a variety of signalling pathways in a broad range of cell types.

**Figure 7:**
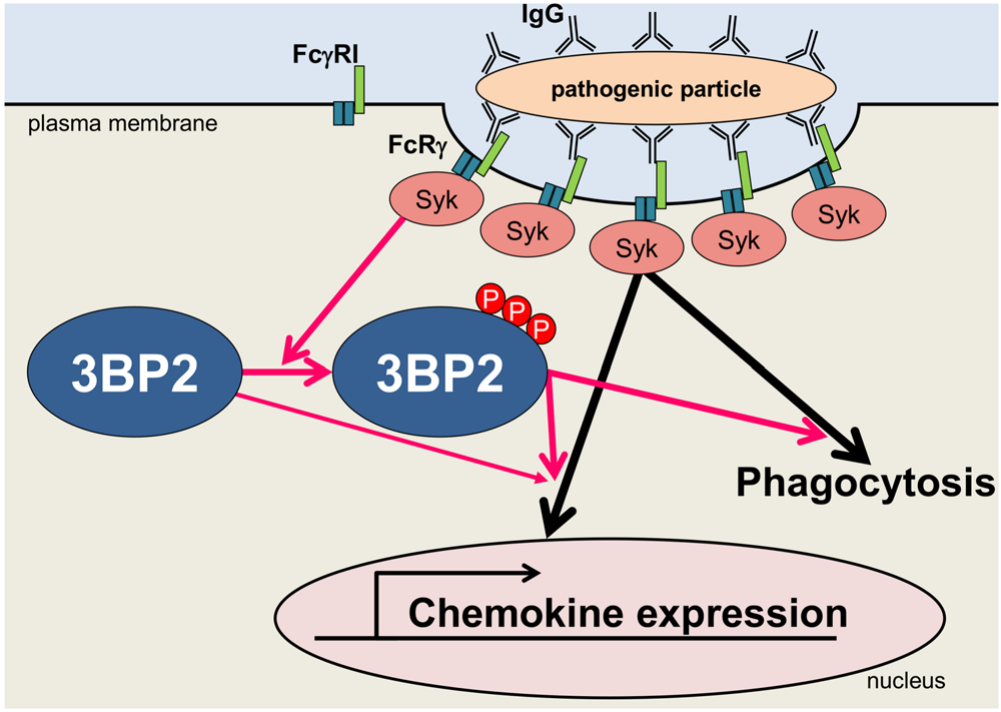
Schematic representation of the role of 3BP2 on FcγR-mediated signalling and the physiological relevance of Syk-dependent phosphorylation. IgG-coated pathogenic particles induce cross-linking of Fc gamma RI (FcγRI), which forms a protein complex with Fc receptor γ (FcRγ) to activate Syk. The activation of Syk triggers various signalling cascades to promote phagocytosis and gene expression. Syk-dependent tyrosine phosphorylation of 3BP2 (P) is critical for optimal FcRγ-mediated phagocytosis and chemokine mRNA expression.

In conclusion, we found that 3BP2 participates in FcRγ-mediated phagocytosis and chemokine mRNA expression. It appears that Syk-dependent tyrosine phosphorylation is critical for the regulation of 3BP2 and the cherubism mutation intricately influences FcγR-mediated signalling (Fig. 7). Nonetheless, it may be important to analyse the impact of Syk-dependent tyrosine phosphorylation of 3BP2 and the cherubism mutation on the clearance of pathogenic particles mediated by phagocytic cells *in vivo* in future studies.

## Materials

### Construction of plasmid DNA for expressing the CRISPR/Cas9 system

To express the CRISPR/Cas9 system in parental U937 cells, a pair of oligo DNAs to generate the single-guide RNA corresponding to each target gene was synthesised, annealed and ligated into *BbsI* sites of the pX330 vector (a gift from Dr. Feng Zhang; Addgene plasmid #42230, MA, USA)^33^. Sequences of the synthetic oligo DNAs are listed online in Supplementary Table S1.

### Cell culture, transfection and establishment of knockout cells

U937 and HL-60 cells were maintained in RPMI1640 containing 10% (v/v) heat-inactivated fetal calf serum. For establishment of the KO cells, 30 μg of the pX330 vector and 10 μg of pEF1α-myc-His A vector containing the G418 resistance gene (ThermoFisher, MA, USA) were electroporated into the U937 cells using a Gene Pulser Xcell (Bio-Rad, CA, USA). G418 (1 mg/ml) resistant clones were selected and expression of the target gene was analysed by immunoblotting. Genomic DNAs were extracted from the selected clones and the mutations generated by the CRISPR/Cas9 system were identified by DNA sequencing as described^53, 54^. For functional analyses, U937 cells were cultured in the presence of 50 ng/ml of IFN-γ (Wako, Osaka, Japan) for 72 h to enhance the cell surface expression of FcγRI^34, 35^.

### Preparation of detergent-soluble cell lysates and immunoblotting analysis

Preparation of detergent-soluble cell lysates and immunoblotting analyses were performed as described^21, 22, 55^. The following antibodies were used in this study: Anti-FcRγ, anti-GAPDH (clone MAB374) and anti-phosphotyrosine (pY)(clone 4G10) were obtained from Merck Millipore (MA, USA); Anti-Syk (clone 4D10), anti-Hck, anti-Abl and anti-3BP2 (clone C-5) were obtained from Santa Cruz Biotechnology (CA, USA). Anti-phosphoSyk (Tyr525/526), anti-phosphoLAT(Tyr191) and anti-LAT antibodies were obtained from Cell Signaling Technology (Danvers, MA).

### Cross-linking of FcγRI and pull-down assay

IFN-γ-stimulated U937 or HL-60 cells (1 × 10^7^) were incubated with 2 μg/ml of mIgG2a (Medical and Biological Laboratories, Aichi, Japan), which is specifically recognised by human FcγRI^36^ for 10 min at 37 °C. Cells were then washed with serum free medium to remove unbound mIgG2a and subsequently stimulated with 10 μg/ml of F(ab′)_2_ fragments of anti-mouse IgG produced in goat (Jackson ImmunoResearch, PA, USA) for 10 min at 37 °C. Cells were then washed with PBS twice and the detergent-soluble cell lysates were prepared. In some experiments, prepared cell lysates were subjected to pull-down assays for analysing tyrosine phosphorylation of 3BP2 induced by the cross-linking of FcγRI. Pull-down assays using GST-Abl-SH3 (residues between 84 to 138 of mouse c-Abl) were performed as described previously^14, 56^.

### Phagocytosis assay

pHrodo Green Zymosan A Bioparticles (ThermoFisher), whose fluorescence is dramatically increased at low pH conditions in mature phagolysosomes, were opsonised by incubation with 50% (v/v) human serum (Sigma-Aldrich, MO, USA) in PBS for 30 min at 37 °C. After extensive washing, opsonised zymosan was incubated with IFN-γ treated cells for 60 min at 37 °C. The fluorescence intensity of the cells was monitored by a FACSCanto II (Beckton Dickinson, NJ, USA). The mean fluorescence intensity and percentage of zymosan positive cells was analysed using FlowJo software (FlowJo LLC, OR, USA).

### Generation of 3BP2-KO cells or HL-60 cells stably expressing HA-3BP2

The cDNA encoding human 3BP2 was amplified from first strand cDNA generated from BJAB cells (a gift from Dr. Satoshi Ishido, Hyogo College of Medicine, Hyogo, Japan). The PCR primer sequences were as follows: 5′-TTGGATCCATGGCGGCTGAAGAGATGCATTG-3′ and 5′-AAGGATCCTCACCTAGGCCCAGTGTAGCCGT-3′. The cDNA encoding mutant form of human 3BP2 of which the PH domain (residues between 1 to 133) is deleted was amplified by PCR using the following forward primer: 5′-GGATCCGAAAAGAAAGACCTGCCCTTGGAC-3′. The sequence of the amplified cDNA was verified and ligated into the pcDNA3.1 HA vector (a gift from Dr. Keiji Tanaka, Tokyo Metropolitan Institutes of Medical Science, Tokyo, Japan) in order to add the HA tag to the N-terminus of 3BP2. The cDNA of HA-tagged 3BP2 was cloned into pEF1α-myc-His A vector. PCR-based site-directed mutagenesis for substitution of Tyr174, 183 and 448 to Phe (HA-3BP2-3F), Pro418 to Arg (HA-3BP2-PR) or Arg488 to Lys (HA-3BP2-RK) was performed using the following primers: Tyr174 to Phe (forward 5′-ACCCCACGGACAATGAAGACTTTGAGCACGACG-3′ and reverse 5′-CGTCGTGCTCAAAGTCTTCATTGTCCGTGGGGT-3′); Tyr183 to Phe (forward 5′-GATGAGGATGACTCCTTCCTGGAGCCTGACT-3′ and reverse 5′-AGTCAGGCTCCAGGAAGGAGTCATCCTCATC-3′); Tyr448 to Phe (forward 5′-CGACTCGGACGAGGACTTTGAGAAGGTGCC-3′ and reverse 5′-GGCACCTTCTCAAAGTCCTCGTCCGAGTCG-3′); Pro418 to Arg (forward 5′-AGCGATCACCCCGCGATGGGCAGAG-3′ and reverse 5′-CTCTGCCCATCGCGGGGTGATCGCT-3′); Arg488 to Lys (forward 5′-GGATGGACTCTACTGCATCAAGAACTCCTCTACCAAGTCG-3′ and reverse 5′-CGACTTGGTAGAGGAGTTCTTGATGCAGTAGAGTCCATCC-3′). To establish 3BP2-KO cells stably expressing HA-3BP2-WT, HA-3BP2-3F or HA-3BP2-PR, 20 μg of each pEF1α-myc-His A vector harbouring one of the three cDNAs and 2 μg of the pApuro vector (a gift from Dr. Tomohiro Kurosaki, Osaka University, Osaka, Japan) containing the puromycin resistant gene was electroporated into the 3BP2-KO cells (3BP2-KO#3). Puromycin (0.5 μg/ml) resistant clones were selected and the expression of HA-3BP2 was analysed by immunoblotting. To establish HL-60 cells stably expressing HA-3BP2-WT, HA-3BP2-dPH or HA-3BP2-RK, 20 μg of each pEF1α-myc-His A vector harbouring one of the three cDNAs was electroporated into HL-60 cells. G418 (1 mg/ml) resistant clones were selected and the expression of HA-3BP2 was analysed by immunoblotting.

### Microarray and real-time PCR analyses

After cells were activated by cross-linking of FcγRI as described above, with the exception of cells that were stimulated for 60 min, total RNA was purified using the High Pure RNA Isolation Kit (Roche, Mannheim, Germany). Microarray and real-time PCR analyses were performed as described previously^53, 54, 57^. Data processing, normalisation, and hierarchical clustering were performed using Subio Platform ver. 1.20 (Subio Inc., Kagoshima, Japan). The following primers were used for real-time PCR: *IL8* (forward 5′-CTCTTGGCAGCCTTCCTGATT-3′ and reverse 5′-TATGCACTGACATCTAAGTTCTTTAGCA-3′); *CCL3L3* (forward 5′-ACAACCGAGTGGCTGTCATC-3′ and reverse 5′-CTTTATTATTTCCCCAGGCCGATC-3′); *CCL4L2* (forward 5′-AAAACCTCTTTGCCACCAATACC-3′ and reverse 5′-GAGAGCAGAAGGCAGCTACTAG-3′); *GAPDH* (forward 5′-TTCACCACCATGGAGAAGGC-3′ and reverse 5′-GGCATGGACTGTGGTCATGA-3′). The mRNA expression levels of *GAPDH* were used for normalisation.

### Statistical analysis

An unpaired two-tailed Student *t*-test was used to assess for statistically significant differences throughout this study.

## Electronic supplementary material

Supplementary information

## References

[1] Gordon S (2016). Phagocytosis: An Immunobiologic Process. Immunity.

[2] Underhill DM, Goodridge HS (2012). Information processing during phagocytosis. Nat. Rev. Immunol..

[3] Takai T (2002). Roles of Fc receptors in autoimmunity. Nat. Rev. Immunol..

[4] Nimmerjahn F, Ravetch JV (2008). Fcgamma receptors as regulators of immune responses. Nat. Rev. Immunol..

[5] Pincetic A (2014). Type I and type II Fc receptors regulate innate and adaptive immunity. Nat. Immunol..

[6] Wang AV, Scholl PR, Geha RS (1994). Physical and functional association of the high affinity immunoglobulin G receptor (Fc gamma RI) with the kinases Hck and Lyn. J. Exp. Med..

[7] Fitzer-Attas CJ (2000). Fcgamma receptor-mediated phagocytosis in macrophages lacking the Src family tyrosine kinases Hck, Fgr, and Lyn. J. Exp. Med..

[8] Mócsai A, Ruland J, Tybulewicz VLJ (2010). The SYK tyrosine kinase: a crucial player in diverse biological functions. Nat. Rev. Immunol..

[9] Greuber EK, Pendergast AM (2012). Abl family kinases regulate FcγR-mediated phagocytosis in murine macrophages. J. Immunol..

[10] Sada K, Takano T, Yanagi S, Yamamura H (2001). Structure and Function of Syk Protein-Tyrosine Kinase. J. Biochem..

[11] Tohyama Y, Yamamura H (2009). Protein tyrosine kinase, syk: A key player in phagocytic cells. J. Biochem..

[12] Gross O (2006). Card9 controls a non-TLR signalling pathway for innate anti-fungal immunity. Nature.

[13] Gross O (2009). Syk kinase signalling couples to the Nlrp3 inflammasome for anti-fungal host defence. Nature.

[14] Ren R, Mayer BJ, Cicchetti P, Baltimore D (1993). Identification of a ten-amino acid proline-rich SH3 binding site. Science.

[15] Deckert M, Tartare-Deckert S, Hernandez J, Rottapel R, Altman A (1998). Adaptor function for the Syk kinases-interacting protein 3BP2 in IL-2 gene activation. Immunity.

[16] Ueki Y (2001). Mutations in the gene encoding c-Abl-binding protein SH3BP2 cause cherubism. Nat. Genet..

[17] Hatani T, Sada K (2008). Adaptor protein 3BP2 and cherubism. Curr. Med. Chem..

[18] Sada K (2002). Regulation of FcεRI-mediated degranulation by an adaptor protein 3BP2 in rat basophilic leukemia RBL-2H3 cells. Blood.

[19] Maeno K (2003). Adaptor protein 3BP2 is a potential ligand of Src homology 2 and 3 domains of Lyn protein-tyrosine kinase. J. Biol. Chem..

[20] Foucault I (2005). The adaptor protein 3BP2 associates with VAV guanine nucleotide exchange factors to regulate NFAT activation by the B-cell antigen receptor. Blood.

[21] Shukla U, Hatani T, Nakashima K, Ogi K, Sada K (2009). Tyrosine phosphorylation of 3BP2 regulates B cell receptor-mediated activation of NFAT. J. Biol. Chem..

[22] Chihara K (2014). Tyrosine phosphorylation of 3BP2 is indispensable for the interaction with VAV3 in chicken DT40 cells. Exp. Cell Res..

[23] Qu X (2005). Tyrosine phosphorylation of adaptor protein 3BP2 induces T cell receptor-mediated activation of transcription factor. Biochemistry.

[24] Jevremovic D, Billadeau DD, Schoon RA, Dick CJ, Leibson PJ (2001). Regulation of NK cell-mediated cytotoxicity by the adaptor protein 3BP2. J. Immunol..

[25] de La Fuente MA, Kumar L, Lu B, Geha RS (2006). 3BP2 deficiency impairs the response of B cells, but not T cells, to antigen receptor ligation. Mol. Cell. Biol..

[26] Chen G (2007). The 3BP2 adapter protein is required for optimal B-cell activation and thymus-independent type 2 humoral response. Mol. Cell. Biol..

[27] Ueki Y (2007). Increased myeloid cell responses to M-CSF and RANKL cause bone loss and inflammation in SH3BP2 ‘cherubism’ mice. Cell.

[28] Mukai T (2014). SH3BP2 cherubism mutation potentiates TNF-α-induced osteoclastogenesis via NFATc1 and TNF-α-mediated inflammatory bone loss. J. Bone Miner. Res..

[29] Levaot N (2011). Loss of Tankyrase-mediated destruction of 3BP2 is the underlying pathogenic mechanism of cherubism. Cell.

[30] Guettler S (2011). Structural basis and sequence rules for substrate recognition by Tankyrase explain the basis for cherubism disease. Cell.

[31] Prod’Homme V (2015). Cherubism allele heterozygosity amplifies microbe-induced inflammatory responses in murine macrophages. J. Clin. Invest..

[32] Yoshitaka T (2014). Enhanced TLR-MYD88 signaling stimulates autoinflammation in SH3BP2 cherubism mice and defines the etiology of cherubism. Cell Rep..

[33] Cong L (2013). Multiplex genome engineering using CRISPR/Cas systems. Science.

[34] Guyre P, Morganelli P, Miller R (1983). Recombinant immune interferon increases immunoglobulin G Fc receptors on cultured human mononuclear phagocytes. J. Clin. Invest..

[35] Celada A, Allen R, Esparza I, Gray PW, Schreiber RD (1985). Demonstration and partial characterization of the interferon-gamma receptor on human mononuclear phagocytes. J. Clin. Invest..

[36] Lubeck MD (1985). The interaction of murine IgG subclass proteins with human monocyte Fc receptors. J. Immunol..

[37] Ainsua-Enrich E (2012). The adaptor 3BP2 is required for early and late events in FcεRI signaling in human mast cells. J. Immunol..

[38] Takai T, Li M, Sylvestre D, Clynes R, Ravetch JV (1994). FcR γ chain deletion results in pleiotrophic effector cell defects. Cell.

[39] Fernández N, Renedo M, García-Rodríguez C, Sánchez Crespo M (2002). Activation of monocytic cells through Fcγ receptors induces the expression of macrophage-inflammatory protein (MIP)-1α, MIP-1β, and RANTES. J. Immunol..

[40] Dai X (2009). Differential signal transduction, membrane trafficking, and immune effector functions mediated by FcγRI versus FcγRIIa. Blood.

[41] Colobran R, Pedrosa E, Carretero-Iglesia L, Juan M (2010). Copy number variation in chemokine superfamily: The complex scene of CCL3L–CCL4L genes in health and disease. Clin. Exp. Immunol..

[42] Agarwal A, Salem P, Robbins KC (1993). Involvement of p72(syk), a protein-tyrosine kinase, in Fcγ receptor signaling. J. Biol. Chem..

[43] Murphy MA (1998). Tissue hyperplasia and enhanced T-cell signalling via ZAP-70 in c-Cbl-deficient mice. Mol. Cell. Biol..

[44] Huang ZY (2011). Interaction of two phagocytic host defense systems: Fcγ receptors and complement receptor 3. J. Biol. Chem..

[45] Levaot N (2011). 3BP2-deficient mice are osteoporotic with impaired osteoblast and osteoclast functions. J. Clin. Invest..

[46] Strzelecka A, Kwiatkowska K, Sobota A (1997). Tyrosine phosphorylation and Fcγ receptor-mediated phagocytosis. FEBS Lett..

[47] Tridandapani S (2000). The adapter protein LAT enhances Fcγ receptor-mediated signal transduction in myeloid cells. J. Biol. Chem..

[48] Bonilla FA, Fujita RM, Pivniouk VI, Chan AC, Geha RS (2000). Adapter proteins SLP-76 and BLNK both are expressed by murine macrophages and are linked to signaling via Fcγ receptors I and II/III. Proc. Natl. Acad. Sci. USA.

[49] Goodridge HS, Simmons RM, Underhill DM (2007). Dectin-1 stimulation by Candida albicans yeast or zymosan triggers NFAT activation in macrophages and dendritic cells. J. Immunol..

[50] Schoenen H (2014). Differential control of Mincle-dependent cord factor recognition and macrophage responses by the transcription factors C/EBPβ and HIF1α. J. Immunol..

[51] Sancho D, Gómez M, Sánchez-Madrid F (2005). CD69 is an immunoregulatory molecule induced following activation. Trends Immunol..

[52] Miah SMS, Hatani T, Qu X, Yamamura H, Sada K (2004). Point mutations of 3BP2 identified in human-inherited disease cherubism result in the loss of function. Genes Cells.

[53] Yamauchi S (2016). STAT1 is essential for the inhibition of hepatitis C virus replication by interferon-λ but not by interferon-α. Sci. Rep..

[54] Honjoh C (2017). Association of C-type lectin Mincle with FcεRIβγ subunits leads to functional activation of RBL-2H3 cells through Syk. Sci. Rep..

[55] Ogi K (2011). Enhancement of B-cell receptor signaling by a point mutation of adaptor protein 3BP2 identified in human inherited disease cherubism. Genes Cells.

[56] Nakashima K (2012). HCV NS5A protein containing potential ligands for both Src homology 2 and 3 domains enhances autophosphorylation of Src family kinase Fyn in B cells. PLoS One.

[57] Kimura Y (2014). Dectin-1-mediated signaling leads to characteristic gene expressions and cytokine secretion via spleen tyrosine kinase (Syk) in rat mast cells. J. Biol. Chem..

