# Supplementary material for: Syk-dependent tyrosine phosphorylation of 3BP2 is required for optimal FcRγ-mediated phagocytosis and chemokine expression in U937 cells
Source: Sci Rep. 2017 Sep 13;7:11480. doi: 10.1038/s41598-017-11915-5 (PMC5597638; doi:10.1038/s41598-017-11915-5)
Supplement: Supplementary file 1 — Supplementary information [file 41598_2017_11915_MOESM1_ESM.pdf]

Supplementary information

The title of the manuscript: Syk-dependent tyrosine phosphorylation of 3BP2 is required for optimal FcRγ-mediated phagocytosis and chemokine expression in U937 cells

The author list: Kazuyasu Chihara, Yuji Kato, Hatsumi Yoshiki, Kenji Takeuchi, Shigeharu Fujieda, Kiyonao Sada

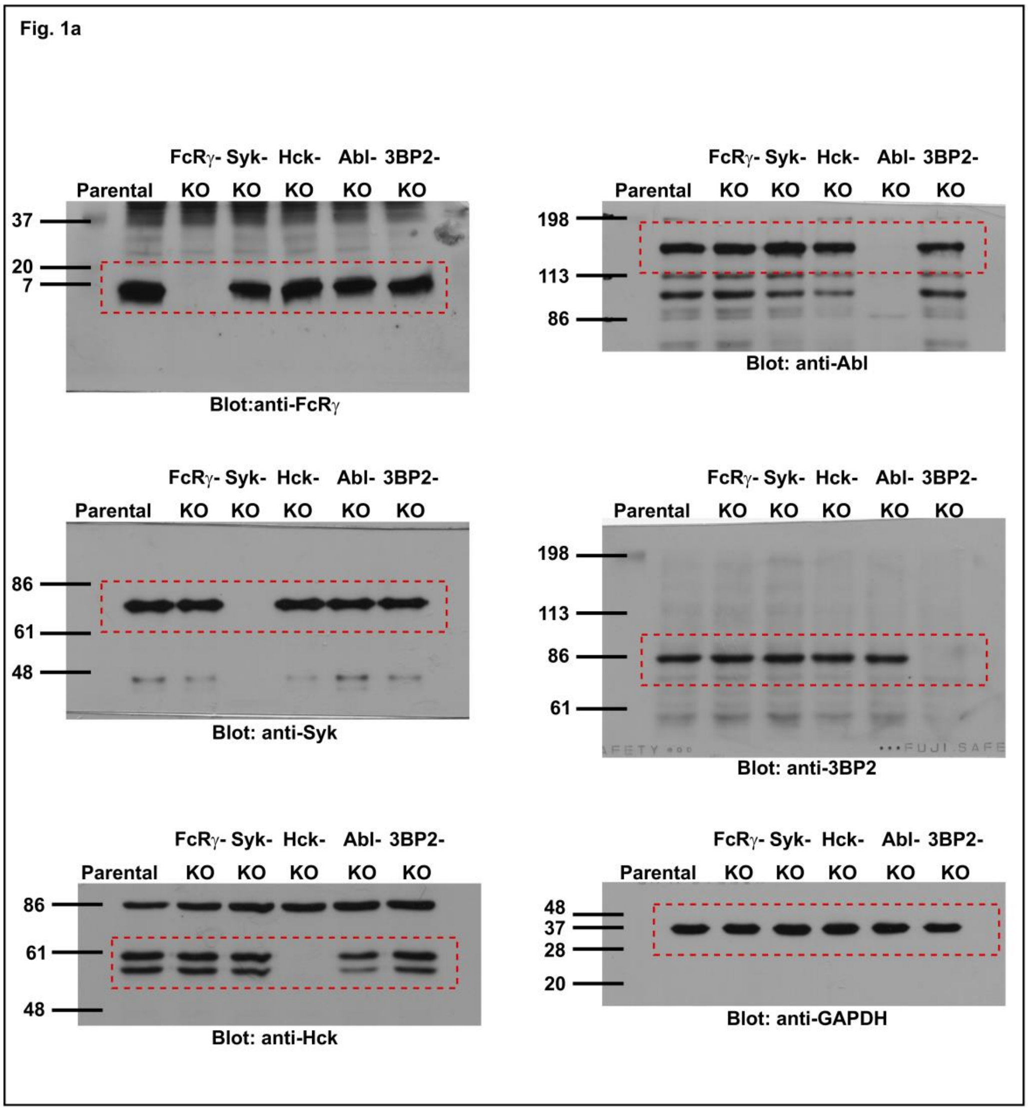

Supplementary Figure S1

Full-length images of immunoblots presented in the main figures

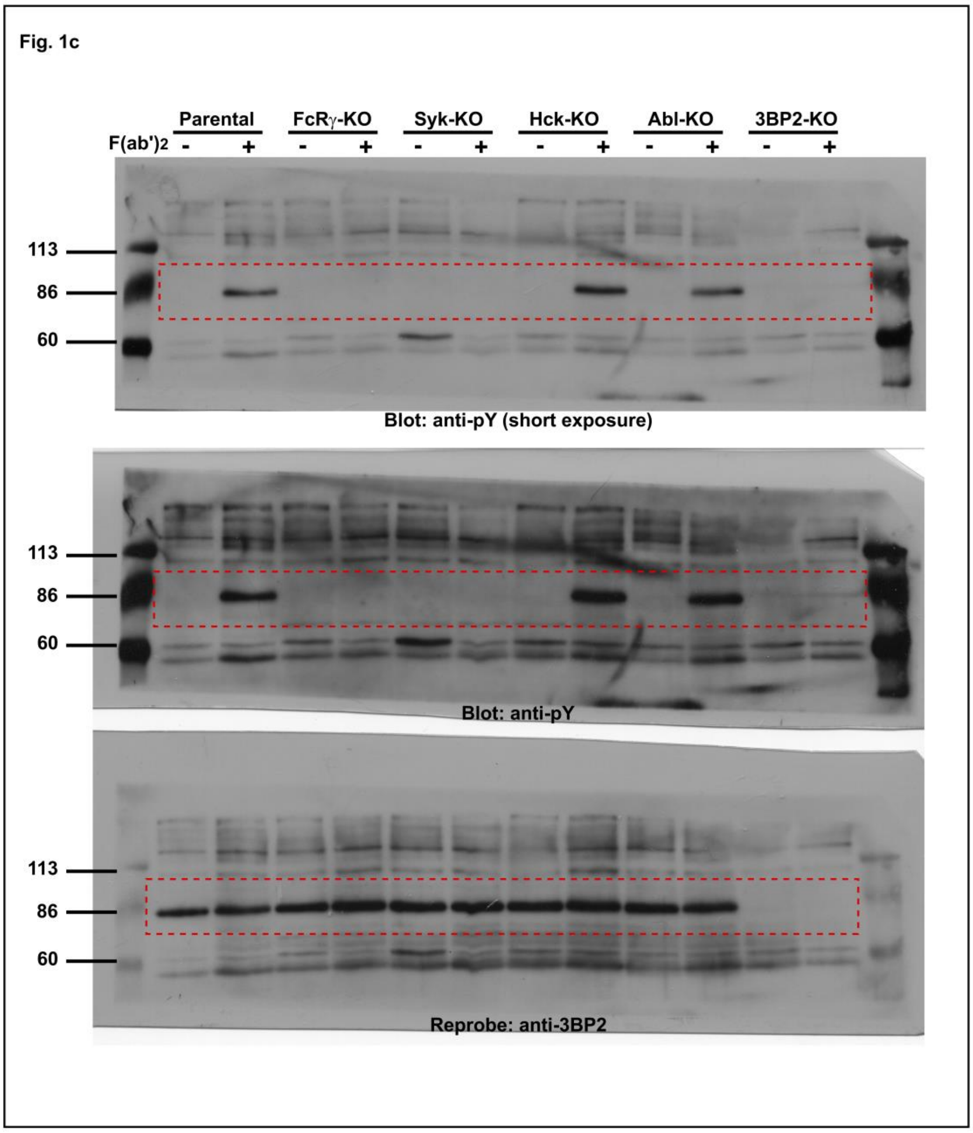

Supplementary Figure S1

Full-length images of immunoblots presented in the main figures

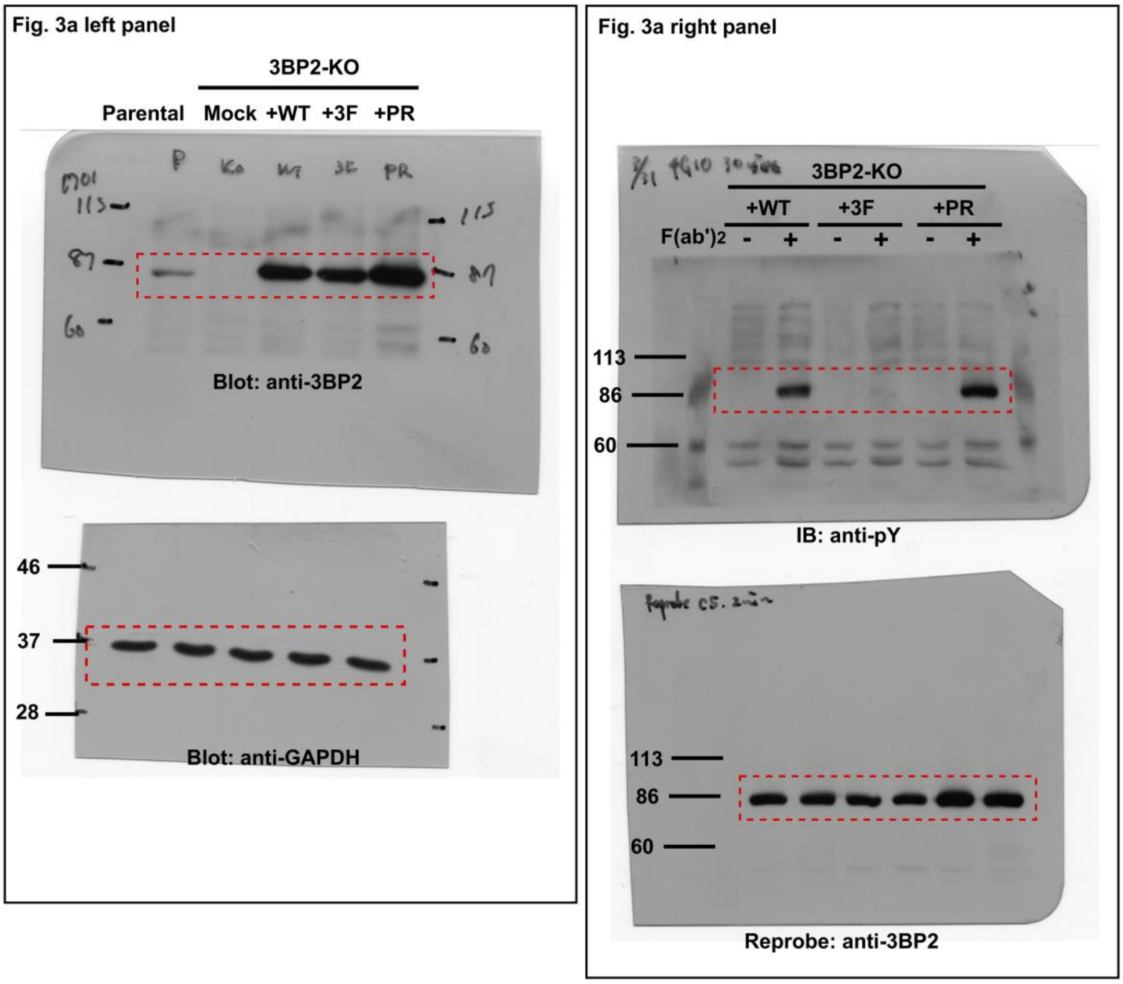

Supplementary Figure S1

Full-length images of immunoblots presented in the main figures

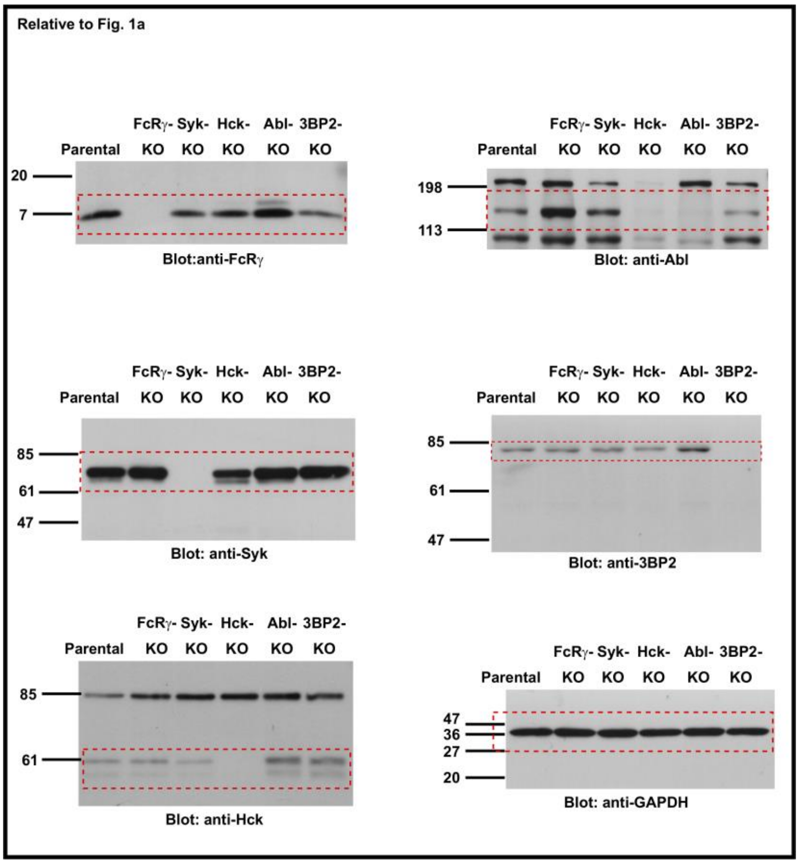

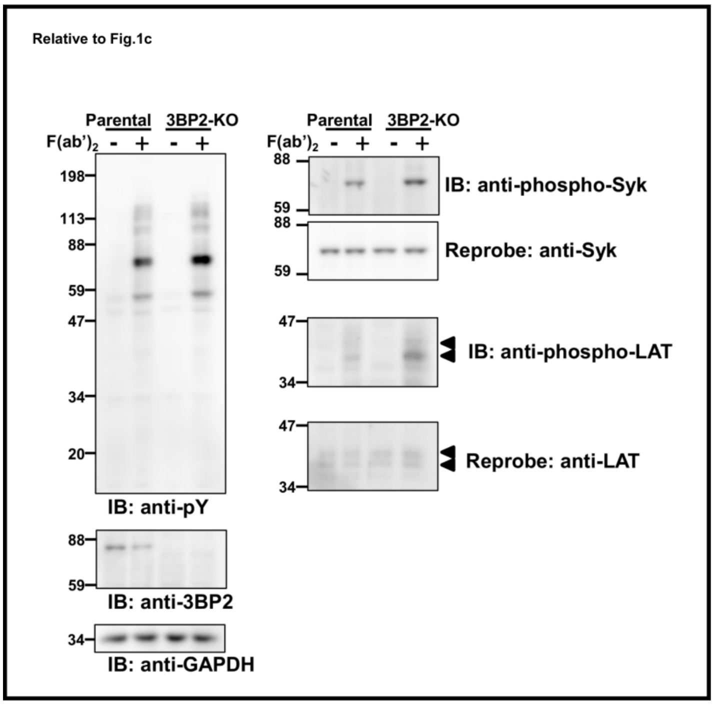

Supplementary Figure S2

Similar results obtained from the other clones

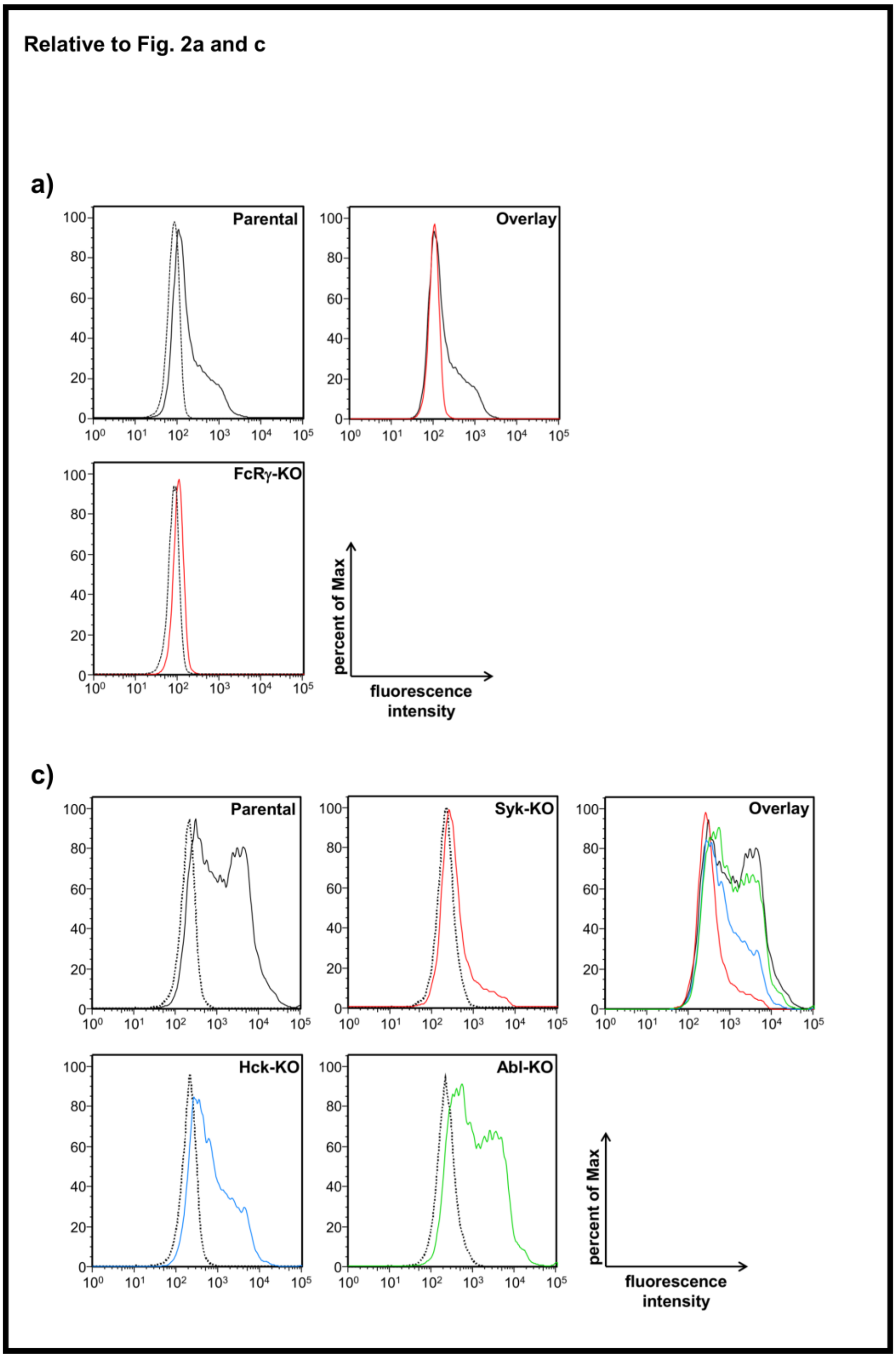

Supplementary Figure S2

Similar results obtained from the other clones

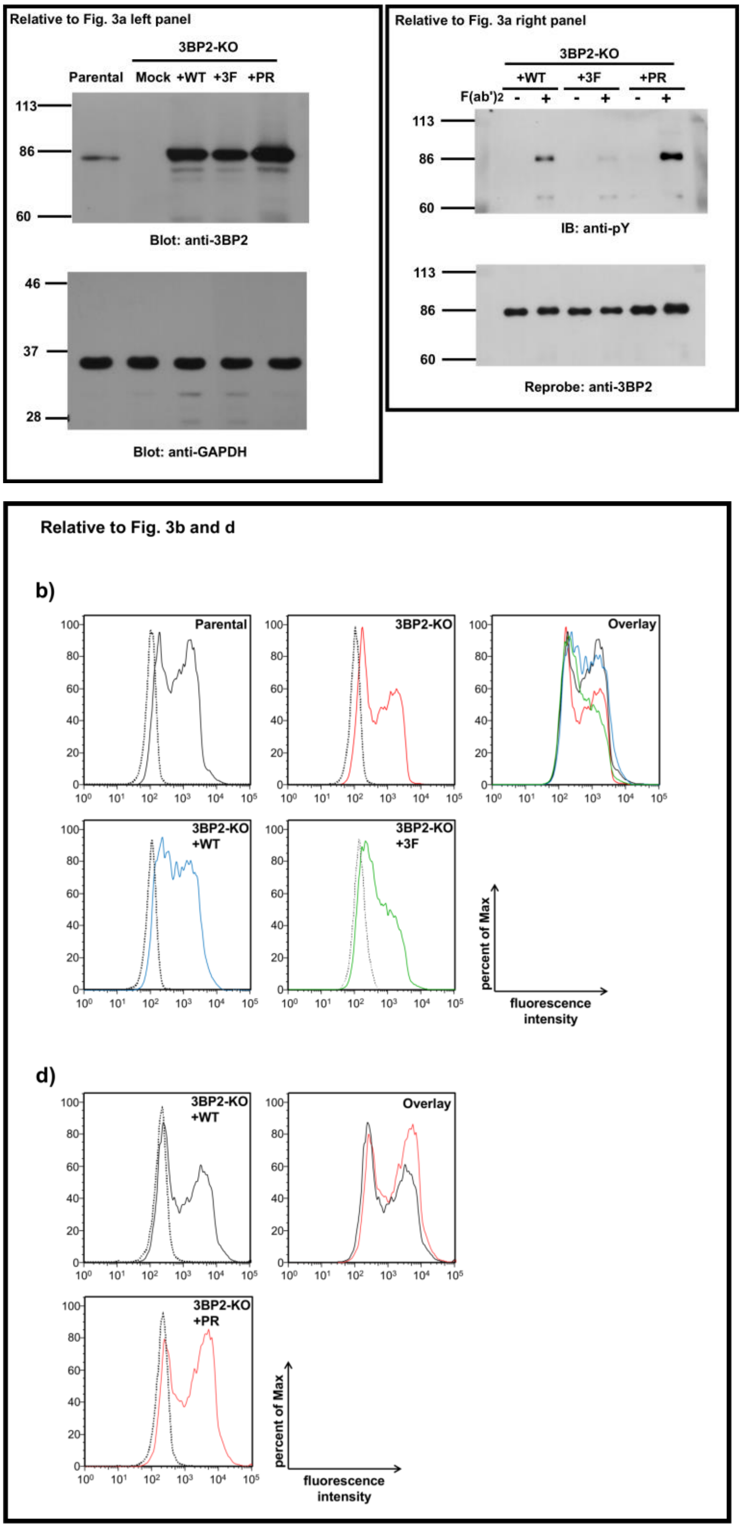

Supplementary Figure S2

Similar results obtained from the other clones

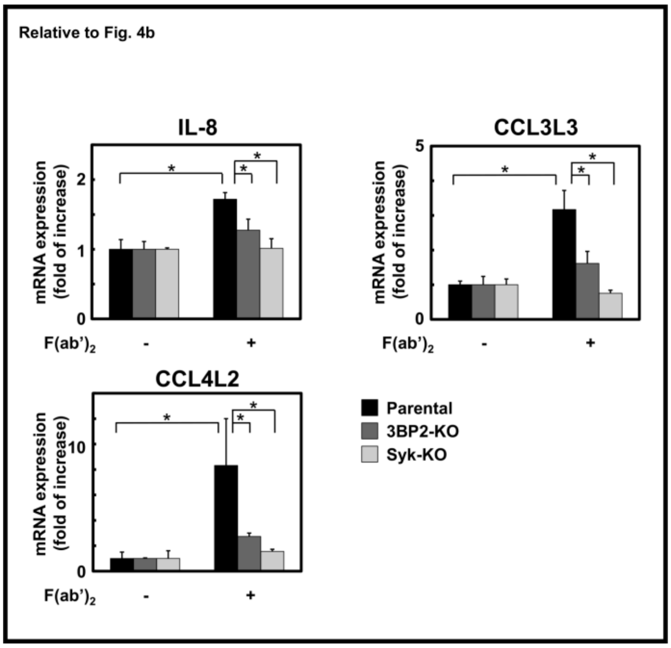

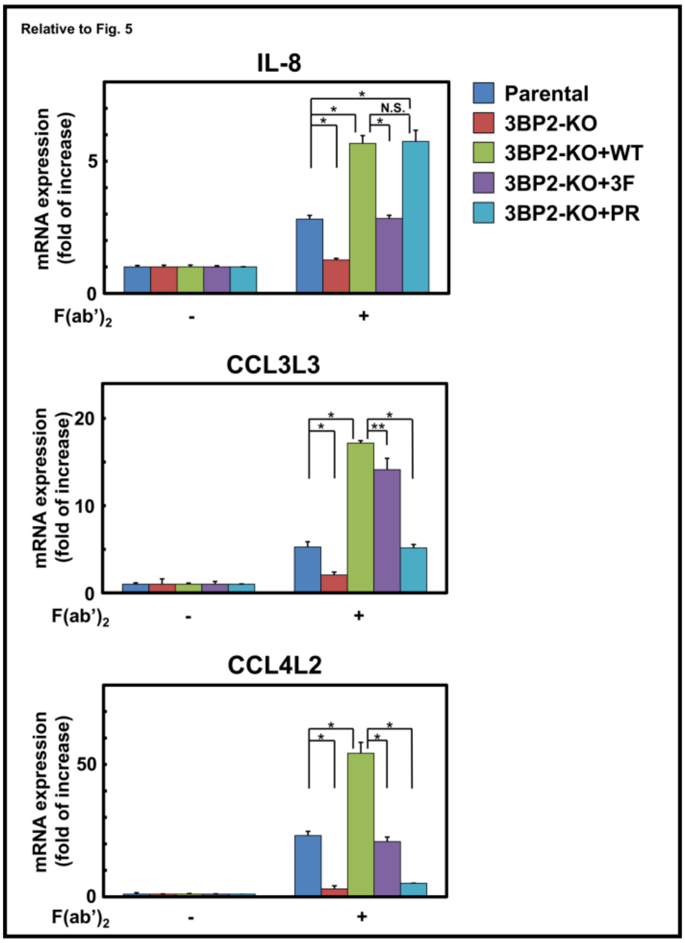

Supplementary Figure S2

Similar results obtained from the other clones

Supplementary Figure S3

Supplementary Materials

Peptide binding experiment was performed as described (Shukla *et al. J. Biol. Chem.*, 2009). In brief, biotinylated peptides corresponding to residues between 166 to 182, 177 to 193 or 438 to 453 of mouse 3BP2 were synthesized (3BP2-peptide Tyr174, 183 and 446 respectively). Peptides containing phosphorylated tyrosine residue corresponding to Tyr174, 183 or 446 of mouse 3BP2 were also synthesized (3BP2 peptide P-Tyr174, 183 and 446 respectively). U937 cell lysates were reacted with these peptides prebound to streptavidin beads. Interaction of the peptides with PLCγ2 and Vav1 were analyzed by immunoblotting. Anti-PLCγ2 and anti-Vav1 antibodies were obtained from Santa Cruz Biotechnology.

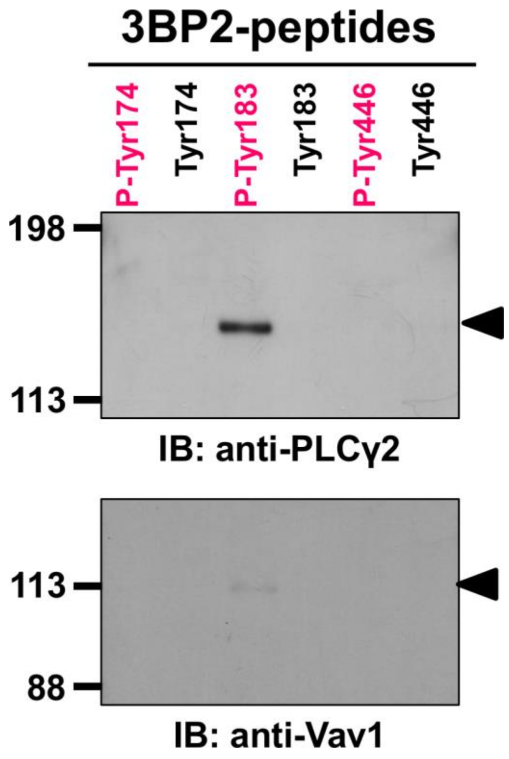

Supplementary Figure S3. Interaction of 3BP2 with PLCγ2 and Vav1.
U937 cell lysates were reacted with the indicated peptides prebound to streptavidin beads. Interaction of the peptides with PLCγ2 and Vav1 were analyzed by immunoblotting. Molecular size markers are indicated on the left in kDa. Data are representative of three independent experiments. Arrowheads indicate the position of PLCγ2 (upper panel) or Vav1 (lower panel).

Supplementary Table S1

Oligo DNA pairs to generate the single-guide RNA for the indicated genes

FcRγ: 5'-CACCGAGTAAGAGCAAGACCACTGC-3'
3'-CTCATTCTCGTTCTGGTGACGCAAA-5'

Syk: 5'-CACCGTTTCGGCAACATCACCCGGG-3'
3'-CAAAGCCGTTGTAGTGGGCCCCAAA-5'

Hck: 5'-CACCGTGGGATCCGGCACGTACAC-3'
3'-CACCCTAGGCCGTGCATGTGCAAA-5'

Abl: 5'-CACCGTCTGAGTGAAGCCGCTCGT-3'
3'-CAGACTCACTTCGGCGAGCACAAA-5'

3BP2: 5'-CACCGGGTACCGCCCTTCTTGTGC-3'
3'-CCCATGGCGGGAAGAACACGCAAA-5'

Supplementary Table S2

Calculated fold changes of FcγRI-induced genes in the indicated cell lines

Affymetrix Human	Cell type→	Parental		3BP2-KO		Syk-KO	
Gene 2.0 ST array	F(ab')2→	-	+	-	+	-	+
Probe set ID	Gene Symbol	Log2 (fold change)		Log2 (fold change)		Log2 (fold change)	
16934764	*ELFN2*	-0.801667	0.248333	0.308333	-0.081666	0.298333	0.028333
17049237	*STAG3*	-0.926667	0.213333	0.083333	0.143333	-0.076667	0.563334
16723653	*CD44*	0.110000	1.390000	0.250000	-0.020000	-1.700000	-0.030000
16989736	*EGR1*	-0.683333	1.006666	-0.463333	0.686667	-0.313333	-0.233333
17075395	*EGR3*	-0.281667	1.338333	-0.531667	0.498333	-0.401667	-0.621667
16833426	*CCL4L2*	-0.298333	0.741667	-0.308333	0.201667	-0.098333	-0.238333
16761201	*CD69*	-0.551667	0.718333	-0.121666	0.338334	-0.451667	0.068333
16689869	*F3*	0.131667	1.461667	-0.368333	0.291667	-0.948333	-0.568334
17035418	*TNF*	0.438333	1.798334	-0.291667	-0.011667	-0.961667	-0.971667
16967771	*IL8*	-0.040000	2.700000	-0.330000	1.340000	-1.660000	-2.010000
17031687	*IER3*	0.335000	1.395000	0.085000	0.635000	-1.115000	-1.335000
16843602	*CCL3L3*	-0.233333	1.226667	-0.093333	0.076667	-0.303333	-0.673333
16901986	*IL1B*	-0.113333	0.936667	-0.293333	-0.203333	-0.123333	-0.203333
